# Dynamic host immune response in virus-associated cancers

**DOI:** 10.1038/s42003-019-0352-3

**Published:** 2019-03-22

**Authors:** Song Cao, Kristine M. Wylie, Matt A. Wyczalkowski, Alla Karpova, Jessica Ley, Sam Sun, R. Jay Mashl, Wen-Wei Liang, Xiaowei Wang, Kimberly Johnson, John F. DiPersio, Hiram Gay, Lee Ratner, Feng Chen, Douglas R. Adkins, Li Ding

**Affiliations:** 1 Department of Medicine, Washington University in St. Louis, St. Louis, MO 63110 USA; 2 McDonnell Genome Institute, Washington University in St. Louis, St. Louis, MO 63108 USA; 3 Department of Pediatrics, Washington University in St. Louis, St. Louis, MO 63110 USA; 4 Siteman Cancer Center, Washington University in St. Louis, St. Louis, MO 63110 USA; 5 Department of Radiation Oncology, Washington University in St. Louis, St. Louis, MO 63110 USA; 6 Department of Biomedical Engineering, Washington University in St. Louis, St. Louis, MO 63110 USA; 7 Brown School Master of Public Health Program, Washington University in St. Louis, St. Louis, MO 63130 USA; 8 Department of Genetics, Washington University in St. Louis, St. Louis, MO 63110 USA

## Abstract

Viruses drive carcinogenesis in human cancers through diverse mechanisms that have not been fully elucidated but include promoting immune escape. Here we investigated associations between virus-positivity and immune pathway alteration for 2009 tumors across six virus-related cancer types. Analysis revealed that for 3 of 72 human papillomavirus (HPV)-positive head and neck squamous cell carcinoma (HNSC) the HPV genome integrated in immune checkpoint genes *PD-L1* or *PD-L2*, driving elevated expression in the corresponding gene. In addition to the previously described upregulation of the PD-1 immunosuppressive pathway in Epstein-Barr virus (EBV)-positive stomach tumors, we also observed upregulation of the PD-1 pathway in cytomegalovirus (CMV)-positive tumors. Furthermore, we found signatures of T-cell and B-cell response in HPV-positive HNSC and EBV-positive stomach tumors and HPV-positive HNSC patients were associated with better survival when T-cell signals were detected. Our work reveals that viral infection may recruit immune effector cells, and upregulate PD-1 and CTLA-4 immunosuppressive pathways.

## Abstract (web summary)

Cao et al. show that human papillomavirus-positive, head and neck squamous cell carcinoma patients are associated with better survival when T-cells are activated. This study suggests that viral infection may recruit immune effector cells and that it may activate PD-1 and CTLA-4 immunosuppressive pathways.

## Introduction

Since the sequencing of the first genomic DNA from a leukemia patient^1^, various studies have identified somatic and germline variants in key cancer genes^2–5^. These genomic biomarkers may aid in therapy selection^6^. Although large-scale sequencing projects such as The Cancer Genome Atlas (TCGA) and the International Cancer Genome Consortium (ICGC) continue to catalog variants across cancer types, only a minority of patients harbor tumors with genomic aberrations associated with sensitivity to targeted therapy.

Complementary to targeted therapy, cancer immunotherapy utilizes the host immune response to kill tumor cells^7,8^. PD-L1 and PD-L2 on tumor cells or antigen-presenting cells suppress T-cell immune response by binding to PD-1 on T-cells^9,10^. To escape attack by immune cells, tumor cells overexpress PD-L1 by gene amplification, utilization of an ectopic promoter, and disruption of 3′ untranslated regions (3′ UTRs)^11^, in addition to PTEN loss-of function^12^ and *EGFR* mutations^13^. Other studies indicate that EGFR mutations are not associated with an increased PD-L1 expression and a better clinical response of PD-L1 immune checkpoint inhibitors^14,15^. Elevated PD-L1 expression creates an immunosuppressive microenvironment that facilitates tumor progression^16^. Anti-PD-1 and anti-PD-L1 immune checkpoint blockades show favorable clinical outcome for treating patients with high PD-1 and PD-L1 expression^17–20^. Another important immune checkpoint pathway involves CTLA-4 and its ligands CD80 and CD86. CTLA-4 serves as a negative regulator of T-Cell activity. The anti-CTLA-4 blockade is also an effective therapeutic strategy to kill tumor cells^21,22^.

Immune infiltration of the tumor microenvironment correlates with improved survival in cancer patients^23,24^. Despite the importance of immune infiltrates and their theoretical associations with viral-positivity^25^, there is no systematic study of associations between virus-positive samples and the immune response except for some limited studies on human papillomavirus (HPV) in head and neck squamous cell carcinoma (HNSC)^26–29^. Our previous large-scale study demonstrated viral positivity in multiple cancer types^30^. Here, using TCGA RNA-Seq data for six virus-associated tumors, we systematically study the associations between virus-positivity and the tumor microenvironment, as measured by expression of *PD-L1*, *PD-L2*, *PD-1*, *CD80*, *CD86*, *CTLA-4*, *Tim-3*, *LAG3*, and *4-1BB* and the prevalence of infiltrating immune cells across multiple types of human cancers. Specifically, we found the enrichment of a T-cell immune signature in HPV-positive and EBV-positive tumors compared to non-viral tumors. The increase of T-cell immune response is associated with a better prognosis in HPV-positive patients. In addition, we found HPV integrations at *PD-L1* and *PD-L2* are associated with high expression of these genes. Higher levels of *PD-L1*, *PD-L2, PD-1, CD80, CD86, CTLA-4*, *Tim-3*, *LAG3*, and *4-1BB* expression were found in Epstein-Barr virus (EBV)-positive stomach adenocarcinoma (STAD) and cytomegalovirus (CMV)-positive colon and rectum adenocarcinoma (COADREAD) tumors compared to virus-negative tumors, providing the rationale for treating virus-positive tumors by anti-PD-1, and anti-CTLA-4 immune therapy. Besides *PD-L1*, *PD-L2, PD-1, CD80, CD86, CTLA-4*, *Tim-3*, *LAG3*, and *4-1BB*, we found the increase of an inducible co-stimulator (ICOS) expression in both HPV-positive head and neck squamous cell carcinoma and EBV-positive stomach adenocarcinoma tumors. ICOS is the immune checkpoint protein, functionally and structurally related to CD28^31^. A positive ICOS signature may indicate a better clinical outcome of anti-CTLA-4 immune therapy in HPV-positive head and neck squamous cell carcinoma and EBV-positive stomach adenocarcinoma patients^32^.

## Results

### Recurrent HPV Integrations at *PD-L1* or *PD-L2* in HNSC

**Fig. 1:**
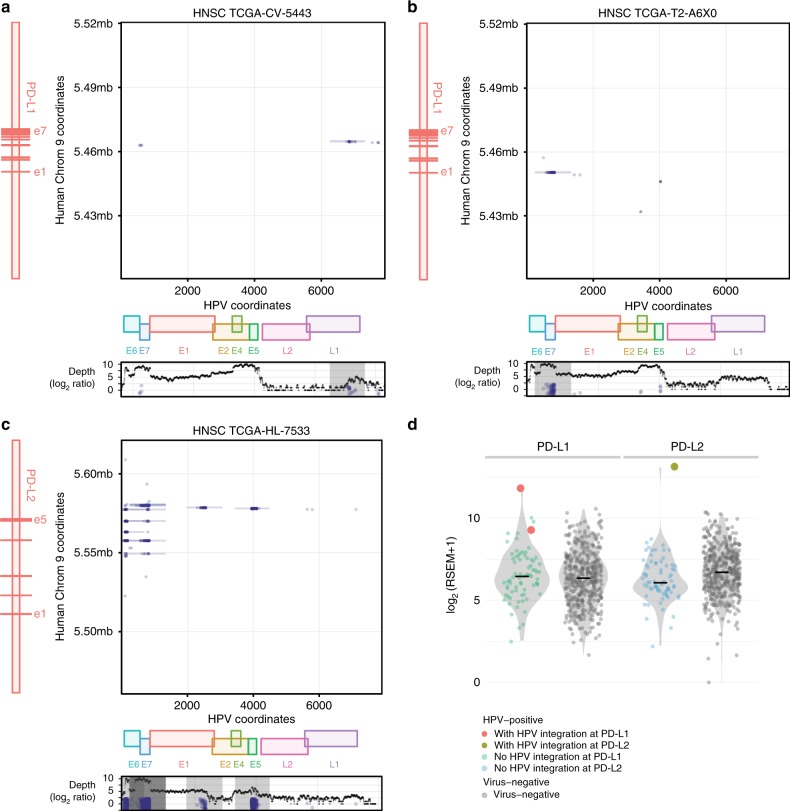
HPV integrations at *PD-L1* and *PD-L2*. **a**–**c** Structural analysis of HPV integration events at *PD-L1* or *PD-L2* for three different HNSC tumors. The dots in the plot show the coordinates in HPV genome (X) and human chromosome (Y) of the breakpoints^67^. The vertical line represents a series of breakpoints clustered together by Pindel^66^. In the figure, e1 represents exon 1. HPV integrates intron 4 and 5′ UTR of *PD-L1* in tumor TCGA-CV-5443 and TCGA-T2-A6X0, respectively. In tumor TCGA-HL-7533, HPV can integrate multiple locations in or after intron 3 of *PD-L2*. The bottom panel shows the number of mapped HPV reads (log_2_ ratio) along with HPV coordinates and *E6*, *E7*, *E1*, *E2*, *E4*, *E5*, *L2*, and *L1* are different genes in HPV genome. **d** The expression of *PD-L1* or *PD-L2* for HPV-positive samples with and without HPV integrations and virus-negative tumors

We analyzed 498 TCGA HNSC tumors using VirusScan and identified 72 HPV-positive tumors (numbers of virus-supporting reads per hundred million reads mapped (RPKM) > 100) and 341 virus-negative tumors (RPKM < 5)^30^. The 413 HNSC tumors with clear HPV status were most common in two ethnicity groups: 364 Caucasians and 30 African Americans. There was no significant difference in HPV status between the two ethnicity groups. We additionally found that HPV-positive HNSC tumors were mostly from males (92%). See Supplementary Table 1. Of these, we identified three tumors with HPV integrations at *PD-L1* or *PD-L2* by using discordant read pair analysis (Methods, Fig. 1a–c). In tumor TCGA-CV-5443, HPV integration sites were localized to intron 4 of *PD-L1*. The same integration site in the same sample was also reported in a previous study^33^. Given a larger cohort size, we also found additional previously unidentified HPV integration sites at *PD-L1* and *PD-L2* in tumors TCGA-T2-A6X0 and TCGA-HL-7533, respectively. Inspection of the detailed discordant read pairs showed that the viral E7 gene integrates into the 5′ UTR region of *PD-L1*; see Fig. 1b. HPV integrations at *PD-L2* appeared more complicated than at *PD-L1*, revealing multiple HPV integration sites in or after intron 3 of *PD-L2*. These three tumors originated in different anatomic sites (larynx, tonsil, and oral cavity). Although different integration patterns and anatomic sites were observed in the three tumors, HPV integrations at *PD-L1* or *PD-L2* were all accompanied by higher expression levels of these genes compared with those in virus-negative tumors (Fig. 1d), and *PD-L1* or *PD-L2* with HPV integrations are expression outliers (see Methods).

To examine the prevalence of HPV integrations at *PD-L1* or *PD-L2* in other tumor types, we analyzed 229 TCGA HPV-positive cervical squamous cell carcinoma and endocervical adenocarcinoma (CESC) tumors and found no putative HPV integrations at either gene. We also checked for integrations of viruses, including HPV, hepatitis B virus (HBV), EBV, and cytomegalovirus, in other cancer types and found no integration events at *PD-L1* or *PD-L2*. This result indicates that high *PD-L1* or *PD-L2* expression induced by HPV integration is a phenomenon that is selectively associated with HPV in HNSC tumors, with *PD-L1* or *PD-L2* integrations occurring in 4.2% of HNSC HPV-positive tumors.

We further looked the relationship between HPV integration and expression in T-cell and B-cell genes. Supplementary Fig. 1 shows the distribution of expression for those genes with HPV integrations. Interestingly, we found that *NR4A2*, *TBC1D1*, *BTNL9*, *DTX1*, *FOXP1*, *INPP4B*, *PDE4D*, and *STAT4* with HPV integration events are expression outliers (see Methods) and all these events are associated with the increase of expression.

### Effect of viral infection on levels of immune checkpoint genes

**Fig. 2:**
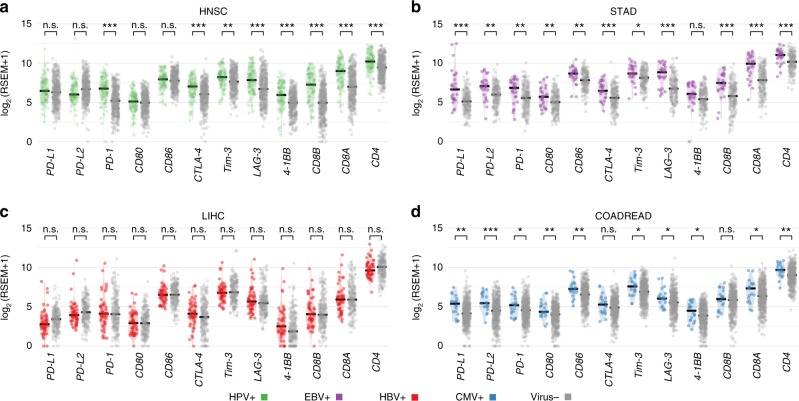
Expression of immune checkpoint genes in virus-positive and negative tumors. The comparison of the expressions of immune checkpoint genes *PD-L1*, *PD-L2*, *PD-1*, *CD80*, *CD86, CTLA-4*, *Tim-3*, *LAG-3*, and *4-1BB* as well as T-cell CD4 and CD8 markers for **a** HPV-positive and virus-negative tumors in HNSC, **b** EBV-positive and virus-negative tumors in STAD, **c** HBV-positive and virus-negative tumors in liver hepatocellular carcinoma and **d** Cytomegalovirus-positive and virus-negative tumors in colon and rectum adenocarcinoma. The “*”, “**”, and “***” symbols indicate *p*-value in the regions [0.05, 0.01], (0.01,0.001], and <0.001, respectively

Having correlated gene-specific viral integration with elevated *PD-L1* and *PD-L2* expression in HPV-positive HNSC, we next correlated any viral infection with *PD-L1*, *PD-L2, PD-1, CD80, CD86, CTLA-4*, *Tim-3*, *LAG3*, and *4-1BB* expressions. In Fig. 2, we compare expression levels of these genes in four tumor types positive for different viruses: HPV in HNSC, EBV in STAD, HBV in liver hepatocellular carcinoma (LIHC), and cytomegalovirus in colon and rectum adenocarcinoma (COADREAD). For the three viruses, we only observed an association between the ethnicity groups for HBV status: 98% are from ASIAN group. We observed most of EBV and HBV tumor patients are Male; See Supplementary Table 1. In HNSC, we found that three tumors with HPV integrations had high expression of *PD-L1* or *PD-L2*, i.e., TCGA-CV-5443 and TCGA-T2-A6X0 with 11.8 and 9.2 for *PD-L1* and TCGA-HL-7533 with 10 for *PD-L2* (RSEM in log2 scale). RSEM stands for RNA-Seq reads by expectation maximization, which is widely used for quantifying gene expression^34^. Overall, no significant difference in *PD-L1*, *PD-L2, CD80*, or *CD86* expression between HPV-positive and virus-negative HNSC tumors was found. A similar observation was also made for HBV (Fig. 2c). However, we found a higher level of *PD-L1*, *PD-L2, CD80, CD86, Tim-3*, *LAG3*, and *4-1BB* in EBV-positive STAD and cytomegalovirus-positive colon and rectum adenocarcinoma than in virus-negative tumor samples (Fig. 2b–d). To leverage the new findings of elevated immune escape pathways of cytomegalovirus-positive colon and rectum adenocarcinoma to other cytomegalovirus-positive tumors, we compared cytomegalovirus-positive and negative tumor samples from stomach and esophageal carcinoma (STES). Supplementary Fig. 2a shows a higher *PD-L1*, *PD-L2*, or *CD80* expression in cytomegalovirus-positive stomach and esophageal carcinoma.

In our previous study, we found a high prevalence of EBV-positive and cytomegalovirus-positive esophageal cancers^30^. In the current study, we found upregulation of *PD-L2* expression (*p* = 0.02), *CD80* (*p* = 0.01), and *CD86* (0.04) associated with EBV or cytomegalovirus positivity (Supplementary Fig. 2b). In this analysis, we combined the EBV and cytomegalovirus-positive tumor samples together to improve the statistical power. In esophageal cancers, we obtained 10 EBV or cytomegalovirus-positive and 89 virus-negative tumors for the statistical analysis. We also noted that *PD-1*, *CTLA-4*, *CD4*, and *CD8* expressions were also higher in the EBV and cytomegalovirus-positive samples, suggesting a higher level of infiltrating T-cells compared with virus-negative tumors (Fig. 2). Although an elevated *PD-L1* expression in EBV-positive samples has been reported in other studies^35,36^, our study shows that EBV or cytomegalovirus infection increases expression of genes encoding *PD-L1*, *PD-L*2, *PD-1, CD80, CD86, CTLA-4, Tim-3*, *LAG3*, and *4-1BB* immune checkpoint genes together with other T-cell markers such as *CD4* and *CD8* in tumors along the gastrointestinal tract, including esophagus, stomach, and intestine.

### Effect of viral infection on host immune response

**Fig. 3:**
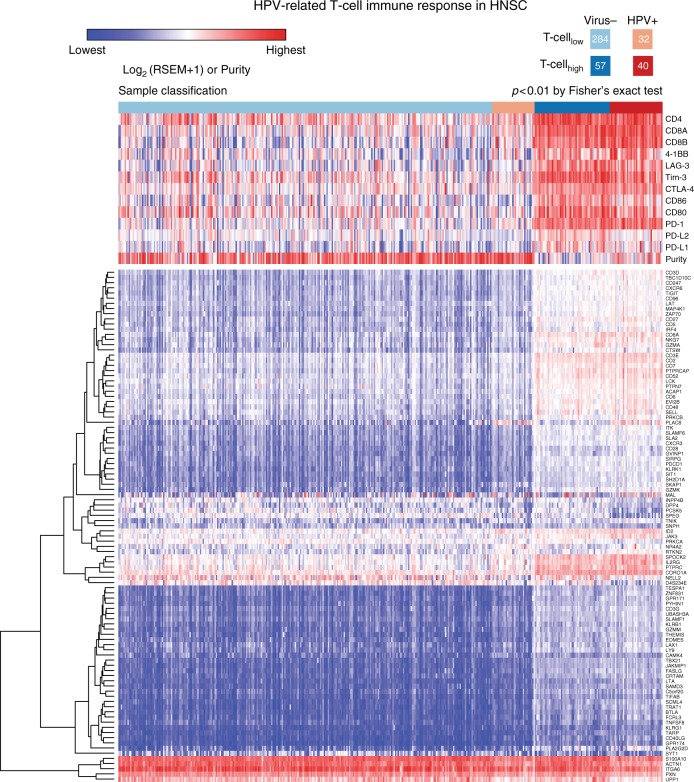
The supervised clustering of T-cell genes in HPV-positive and virus-negative HNSC tumors. In the upper panel, we show the expression of T-ell markers such CD4, CD8 and immune checkpoint genes *PD-L1*, *PD-L2*, *PD-1*, *CD80*, *CD86*, *CTLA-4*, *Tim-3*, *LAG-3*, and *4-1BB*. Tumors are clustered to four different groups based on the T-cell and virus statuses, i.e., Virus-/T-cell_low_, HPV+/T-cell_low_, Virus-/T-cell_high_, and HPV+/T-cell_high_ from left to right

CD4+ and CD8+ T-cells and B-cells play important roles in fighting infection and cancer. Immune infiltration is frequently observed in solid tumors, and is associated with improved host survival^23^. Here, we evaluated associations between viral infection and immune cell infiltration in the tumor microenvironment. We collected a list of genes corresponding to T-cell and B-cell signatures (Supplementary Table 2) from previous publications^37–39^. We then identified 99 genes with significant differential expression (FDR < 0.05, see Methods) between HPV-positive and virus-negative HNSC tumors (Supplementary Data 1, Fig. 3). In the 99 selected genes, we also required that the difference of median values of gene expression (log2) is larger than 1 between the two cohorts. Overall, HPV-positive tumors displayed higher levels of T-cell signatures than virus-negative tumors (Fig. 3). In Fig. 3, we separated samples into four different groups based on supervised clustering results, i.e., Virus-/T-cell_low_, HPV+/T-cell_low_, Virus-/T-cell_high_, and HPV+/T-cell_high_. Overall, HPV-positive tumors displayed higher levels of T-cell signatures than virus-negative tumors (Fig. 3). The expressions of 99 T-cell genes in HPV-positive HNSC tumor are higher than the values in virus-negative samples (Supplementary Fig. 3). GSEA^40,41^ also shows the enrichment of T-cell gene set in HPV-positive HNSC tumors (Supplementary Fig. 4a). By using gene oncology (GO) database^42,43^, we found that most of the 99 genes are classified as gene sets related to immune response, lymphocyte and leukocyte, indicating infiltrated immune cells, and etc. No obviously different clusters were observed in term of GO annotation for these genes (Supplementary Fig. 5). We found that tumors with high T-cell signatures from our clustering method were associated with high *PD-L1*, *PD-L2*, *PD-1*, *CD80*, *CD86*, *CTLA-4*, *Tim-3*, *LAG3*, *4-1BB*, *CD8*, and *CD4* expression. These tumors also associated with high immune scores and lower tumor purity, indicative of a high level of immune infiltration (Supplementary Fig. 6a, c). Tumor purity and immune score were calculated based on the method used by Aran et al.^44^. In addition, HPV-positive HNSC tumors had elevated levels of *PD-1*, *CTLA-4*, *CD8*, and *CD4* compared to virus-negative tumors (Fig. 2a). Similarly, we found an enrichment of B cell signatures in HPV-positive HNSC tumors (Supplementary Figs. 4b, 7).

**Fig. 4:**
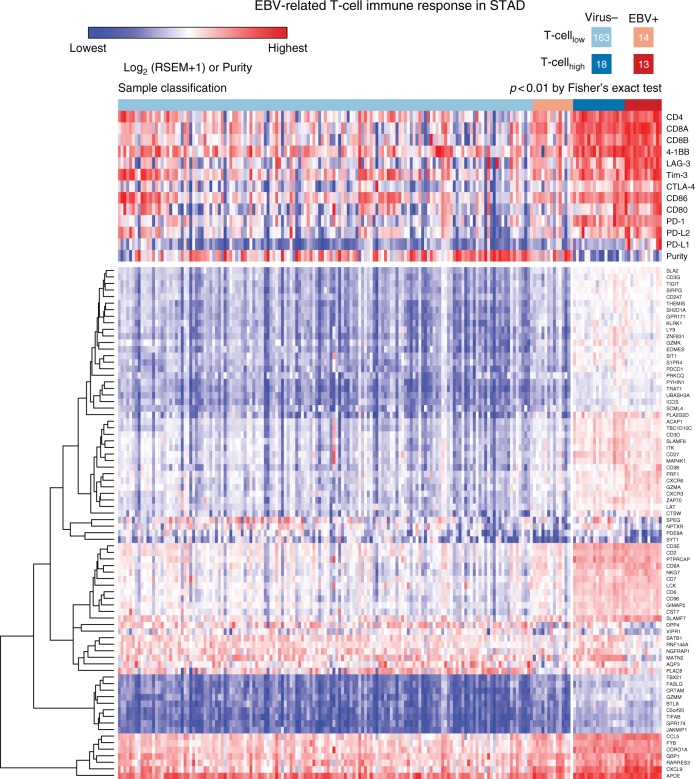
The supervised clustering of T-cell genes in EBV-positive and virus-negative STAD tumors. In the upper panel, we show the expression of T-cell markers such CD4, CD8 and immune checkpoint genes *PD-L1*, *PD-L2*, *PD-1*, *CD80*, *CD86*, *CTLA-4*, *Tim-3*, *LAG-3*, and *4-1BB*. Tumors are clustered to four different groups based on the T-cell and virus statuses, i.e., Virus-/T-cell_low_, EBV+/T-cell_low_, Virus-/T-cell_high_, and EBV+/T-cell_high_ from left to right

**Fig. 5:**
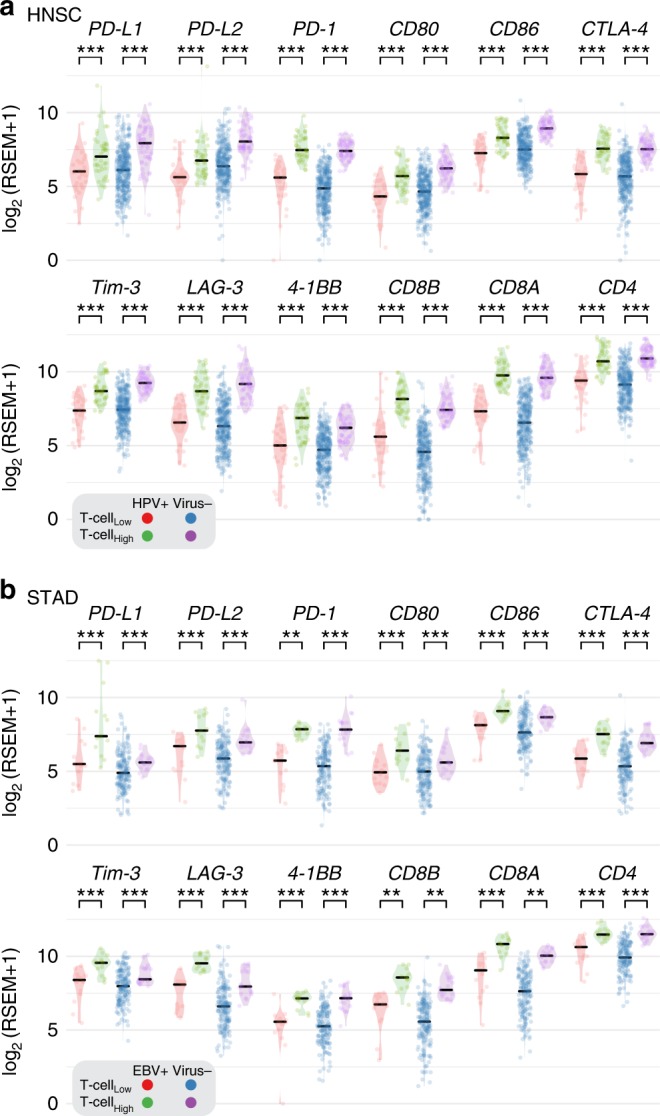
Expression of immune checkpoint genes in T-cell_high_ and T-cell_low_ tumors. The comparison of the expressions of immune checkpoint genes *PD-L1*, *PD-L2*, *PD-1*, *CD80*, *CD86*, *CTLA-4*, *Tim-3*, *LAG-3*, and *4-1BB* as well as T-cell CD4 and CD8 markers between T-cell_high_ and T-cell_low_ tumors in HPV-positive HNSC, EBV-positive STAD and virus-negative cohorts

Next, we identified 78 T-cell genes with significant differential expression (FDR < 0.05, see Methods) between EBV-positive and virus-negative STAD tumors (Fig. 4 and Supplementary Data 1). In the 78 selected genes, we also required that the difference of median values of gene expression (log2) is larger than 1 between the two cohorts. GESA analysis indicates an enrichment of T-cell in EBV-positive STAD tumors (Supplementary Fig. 4c). GO database annotation shows that 77 differential expression are mostly related to immune response, lymphocyte, cell activation, etc. and we did not observe significant different clusters among these genes in term of GO annotation (Supplementary Fig. 8). Tumors with high T-cell signatures from our clustering analysis are concordant with high expression of *PD-L1*, *PD-L2*, *PD-1*, *CD80*, *CD86*, *CTLA-4*, *Tim-3*, *LAG3*, *4-1BB*, *CD8*, and *CD4*, EBV-positive status (Fig. 5), and low tumor purity and high immune score (Supplementary Fig. 6b, d). B-cell response signatures were also observed in EBV-positive STAD tumors based on differential expression of 34 genes compared with non-viral tumors (Supplementary Fig. 9, 4d). This includes human leukocyte antigen (*HLA*) genes such as *HLA-DQB1*, *HLA-DQA1*, *HLA-DMA*, *HLA-DMB*, *HLA-DRB5*, and *HLA-DOA*. These genes play an important role for the formation of major histocompatibility complex (MHC) class II/peptide complex, which recognizes microbial antigens and cancer neoantigens^45^.

From our analyses, 78% of T-cell signature genes in EBV-positive tumors overlapped with those from HPV-positive HNSC, indicating that similar T-cell immune responses are associated with EBV and HPV. For instance, an ICOS signature was identified in EBV-positive STAD (Supplementary Table 2) as well as HPV-positive HNSC; The expression of *ICOS* increases about two-fold in EBV-positive STAD and HPV-positive HNSC compared to virus-negative samples. *ICOS* is an immune checkpoint gene in *CD28* and *CTLA-4* family, which plays important role in regulating the immune response and enhances the antitumor immune response in anti-CTLA-4 blockade^31,32^.

Furthermore, we selected genes, which showed at least four-fold difference in the relative expression between HPV-positive and EBV-positive samples compared to the corresponding virus-negative cohorts. In the T-cell gene list, six genes (*PLAC8*, *CXCL9*, *SATB1*, *PDE9A*, *NPTXR*, and *NELL2*) passed the cut-off. We note that for *PLAC8*, a gene that is associated with pancreatic cancer progression^46^, was highly elevated in HPV-positive samples with about 16-fold increase compared to the virus-negative HNSC samples. However, in EBV-positive STAD sample, the expression of *PLAC8* was downregulated by about four-fold compared to virus-negative STAD sample (Supplementary Table 2). In the B-cell gene list, we identified an additional 18 genes fitting these criteria, such as *STAG3*, *MET1*, *CDKN2A*, *COCH*, *BTNL9*, *SPIB*, *MS4A1*, *CD19*, *CR2*, *BLK*, *VPREB3*, *CXCR5*, *MIR600HG*, *BANK1*, *F5*, *BACH2*, *KYNU*, and *SLC22A3*. *CDKN2A* showed distinct expression alteration in HPV-positive and EBV-positive samples. In HPV-positive samples, the expression of *CDKN2A* was 16-fold higher than virus-negative HNSC samples, but in EBV-positive samples, its expression was three-fold lower than the virus-negative samples. *CDKN2A* is a tumor suppressor, which is highly mutated in virus-negative HNSC samples^47^, but not in HPV-positive HNSC samples. The low expression of *CDKN2A* found in EBV-positive STAD and HPV-negative HNSC samples reflect two different mechanisms inactivating the gene function by EBV infection and somatic mutation. We note that CDKN2A is mostly involved in the regulation of cell cycle^48^, not necessarily related to the immune infiltration. The latter is mainly associated with a low tumor purity and increased overall expression of T-cell genes (Figs. 3, 4). In contrast to HPV-positive HNSC and EBV-positive STAD, we did not find significant enrichment of T-cell and B-cell signatures in HBV-positive liver hepatocellular carcinoma. For colon and rectum adenocarcinoma, we find higher levels of *CD4*, *CD8A*, and *PD-1* in cytomegalovirus-positive tumors than in virus-negative tumors (Fig. 2), though other T-cell genes only showed modest differential expression in cytomegalovirus-positive and virus-negative tumors.

We further compared the T-cell differentiation phenotype in virus-positive and negative tumors for different viruses by using markers such as CD28, CD27, CD45, CD103, perforin, GMP-17, and granzymeA^49^ (see Supplementary Fig. 10). High expression of CD28 and CD27 in HPV-positive HNSC and cytomegalovirus-positive colon and rectum adenocarcinoma tumors suggest an increase in the presence of CD28+CD27+ T-cells compared to virus-negative HNSC tumors and virus-negative colon and rectum adenocarcinoma tumors. CD28 and CD27 are markers for precursor or early differentiation T-cells^49^. The HPV-positive HNSC tumors and cytomegalovirus-positive colon and rectum adenocarcinoma tumors also had higher expression of NK T-cell markers (perforin, GMP-17, and granzymeA) and CD45 compared with virus-negative HNSC and colon and rectum adenocarcinoma tumors. Also, HPV-positive HNSC tumors and EBV-positive tumors had a higher expression of CD103, a marker for resident T-cells, compared to virus-negative samples, but not in cytomegalovirus-positive colon and rectum adenocarcinoma and HBV-positive liver hepatocellular carcinoma tumors.

### Clinical relevance of virus-associated immune response

**Fig. 6:**
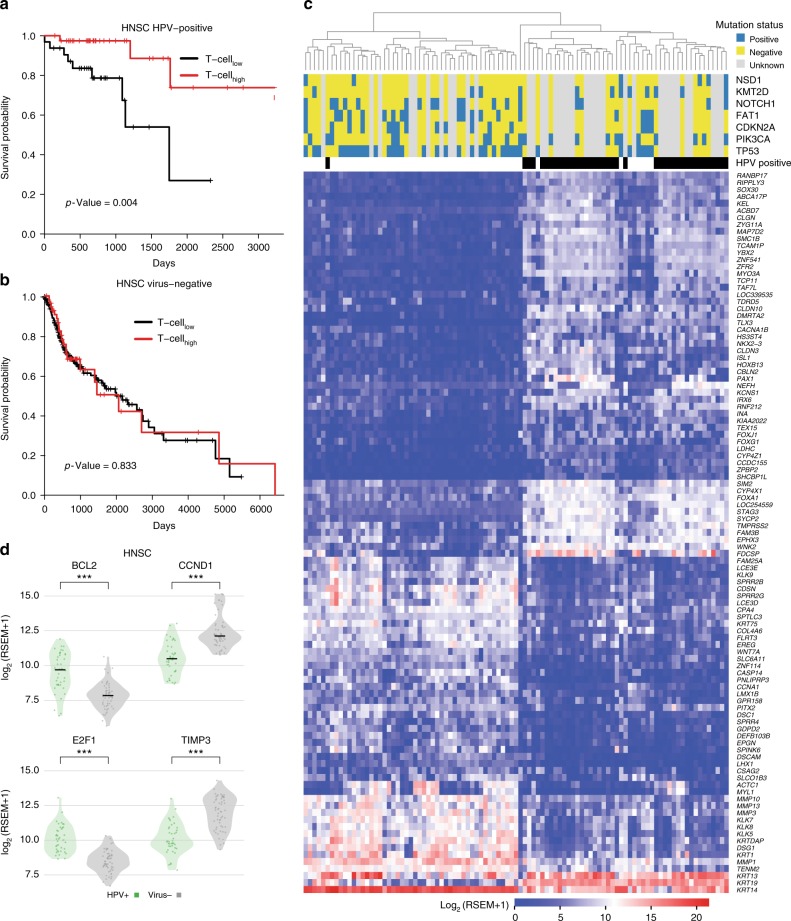
Survival and gene expression analyses in HPV-positive and negative tumors. The comparison of overall survival in T-cell_high_ and T-cell_low_ tumors between two different cohorts: **a** HPV-positive and **b** virus-negative. **c** The mutational status for seven mutated genes in HNSC and the supervised clustering based on 103 genes which demonstrate strong significantly differential expression among HPV-positive and virus-negative HNSC tumors (FDR < 0.05, see Methods). **d** The differentially expressed genes involved in p53 signaling pathway between HPV-positive and virus-negative tumors

Based on our clustering analysis, HPV-positive tumors were more likely to have an elevated immune response (40 of 72 tumors, 55%) compared with virus-negative tumors (57 of 341 tumors, 16%) (*p* < 0.01, Fisher’s exact test, two-tailed) (Fig. 3). We first performed survival analysis to evaluate how immune response correlates with clinical outcome in HPV-positive and virus-negative cohorts (Fig. 6a, b, respectively). We found that immune response is associated with a positive prognosis in patients with HPV-positive HNSC, but not in those with virus-negative HNSC (Fig. 6a). We then examined mutational and gene expression patterns in two cohorts: HPV-positive HNSC with elevated immune response and virus-negative HNSC with elevated immune response (Fig. 6c); in total, we found 2695 genes with at least two-fold differential expression (FDR < 0.05). We identified 103 genes with 16-fold or higher differential expression (Fig. 6c). Notably, we found that the expression of FOXA1, a gene associated with better survival in breast cancer^50^, is about 16-fold higher in HPV-positive tumors. We also identified seven highly mutated genes (*TP53*, *PIK3CA*, *CDKN2A*, *FAT1*, *NOTCH1*, *KMT2D*, and *NSD1*) with frequency greater than 10% in HNSC tumors with elevated immune response. The HPV-positive cohort contained no variants in either *TP53* or *CDKN2A* and only one in *FAT1*; in the HPV-negative cohort, these genes were frequently mutated. HNSC tumors with wild-type *TP53* are more sensitive to radiation therapy than tumors with *TP53* mutations^51^. In addition, we examined differentially expressed genes in the p53 signaling pathway between the two cohorts and found 4 of 16 p53 pathway genes showing substantial expression alteration (see Fig. 6d). Levels of B-cell CLL/lymphoma 2 (BCL2) and E2F transcription factor 1 (E2F1) are higher in HPV-positive tumors. Previous studies show favorable prognosis with a high expression of BCL2^52^ and poor prognosis with overexpressed CCND1^53^. The distinct expression pattern of p53 signaling pathway genes may also drive different clinical outcome of the two cohorts, though both cohorts are associated with an infiltrated immune cell microenvironment.

## Discussion

In this study, we systematically investigated associations between virus infection or integration and alteration of the tumor microenvironment. We found a significant difference (*p* = 0.01, Fisher’s exact test) between HPV integration status at *PD-L1* or *PD-L2* in HPV-positive HNSC (*N* = 72) and CESC (*N* = 229) tumors. Specifically, we found three integrations among the HNSC samples with high expression of PD-L1 or PD-L2 and no integrations among the CESC samples. It is likely that HPV has co-evolved to target *PD-L1* or *PD-L2* to create an immunosuppressive tumor microenvironment in some head and neck cancers. Further investigation with a large sample set is important for leveraging the observed relationship between virus integration at PD-L1 or PD-L2 in HNSC and the increased expression. We also found samples with HPV integrations in other immune-related genes (*NR4A2*, *TBC1D1*, *BTNL9*, *DTX1*, *FOXP1*, *INPP4B*, *PDE4D*, and *STAT4*) have an increased expression of these genes. Previous studies show that high expression of *NR4A2*, *BTNL9*, *FOXP1*, or *PDE4D* can antagonize immune response or is associated with tumor progression^54–57^. In EBV-positive STAD, cytomegalovirus-positive colon and rectum adenocarcinoma and EBV or cytomegalovirus positive stomach and esophageal carcinoma, we found that viral infection associates with high expression of *PD-L1* or *PD-L2*, *CD80*, *CD86, PD-1, CTLA-4, Tim-3*, *LAG3*, and *4-1BB* without integrating into the human genome. Moreover, our study indicates that EBV and cytomegalovirus elevate *PD-L1* or *PD-L2*, *CD80*, *CD86, PD-1, CTLA-4, Tim-3*, *LAG3*, and *4-1BB* expression in multiple cancers along the gastrointestinal tract including stomach and esophageal carcinoma and colon and rectum adenocarcinoma.

A previous study^58^ demonstrated elevated PD-L1 expression in both tumor and immune cells across a large number of tumor samples by using immunohistochemistry (IHC) assays. For example, in 101 head-neck tumors, they found 28 and 19% of immune cell and tumor cell, respectively, were positive for PD-L1 in their samples. The anti-PD-L1 antibody works well on PD-L1 positive tumors to neutralize PD-L1 and make the tumor susceptible to attack by the immune system^58^. Our studies show an elevated *PD-L1* expression in EBV and cytomegalovirus-positive samples, suggesting clinical trials of PD-L1 immunotherapy in these patients may be beneficial. Also, in a subset of HPV-positive HNSC, *PD-L1* is highly expressed when HPV integrates into the *PD-L1*, suggesting these patients may have responded to anti-PD-L1 therapies. The previous clinical trial on HNSC has shown longer overall survival in both HPV-positive and PD-L1-positive tumors when treating with ant-PD-1 monoclonal antibody than the standard single-agent therapy^59^. Furthermore, we found that the expression of *PD-L2, CD80, CD86*, and *CTLA-4* are also elevated in cytomegalovirus and EBV-positive tumor patients, suggesting anti-PD-L2 and anti-CTLA4 immunotherapy may be effective in patients with these types of tumors.

In contrast to the clear association between EBV and stomach adenocarcinoma, some controversy exists about the association between cytomegalovirus and colorectal cancer^60–62^. In our previous large-scale study, we found a higher abundance of cytomegalovirus in tumors than in adjacent normal samples^30^. In the current study, we discovered a high level of *PD-L1*/*PD-L2* in cytomegalovirus-positive tumors across the gastrointestinal tract suggesting that cytomegalovirus mediates the tumor microenvironment, which helps tumor cells to avoid the attack of immune cells.

In addition, we found distinct immune responses for different viruses in different cancer types. A high level of immune response was observed in HPV-positive HNSC and EBV-positive STAD samples but not in HBV-positive liver hepatocellular carcinoma. One explanation is that HBV promotes cancer in a different way than EBV/HPV, which are directly oncogenic, HBV promotes cancer by making the liver cirrhotic/inflamed chronically. The immune response was measured by gene expression of characteristic T-cell and B-cell markers, including CD4, CD8, and PD-1 T-cell markers, and tumor purity, which when low indicates high immune cell infiltration into the tumor microenvironment. We also observed high expression of *ICOS* and *CTLA-4* in both HPV-positive HNSC and EBV-positive STAD, suggesting these tumors may have had an effective clinical response to anti-CTLA-4 immune therapy. Survival analysis shows high immune response is associated with favorable survival in HPV-positive but not HPV-negative HNSC samples. The different mutational status and expression patterns may lead to different clinical outcomes of the immune response. For instance, we found different expression alteration in key genes involved p53 signaling pathway in two cohorts. The complete retention of wild-type TP53 in HPV-positive HNSC tumors is another key factor driving the difference, as previous studies show better radiotherapy sensitivity in HNSC patients with wild-type TP53^51^. For cervical squamous cell carcinoma and endocervical adenocarcinoma (CESC), we separated tumors into low and elevated immune infiltration cohorts according to HPV-positive T-cell signatures (Supplementary Fig. 11). Although tumors with elevated immune response show a better survival rate before eight years, there is no significant difference in overall survival rate based on immune response (Supplementary Fig. 12). Further clustering samples based on a CD8+ T-cell gene list show improved association survival rate and CD8+ T-cell status and patients with CD8+ T-cell status have a higher chance of a tumor-free status (Supplementary Figs. 13, 14).

Our study highlights the importance of viral integration and infection in shaping tumor microenvironments. The current study is necessarily based on gene expression data from RNA-Seq. Proteomics data from the Clinical Proteomic Tumor Analysis Consortium (CPTAC) will enable us to investigate the virus-mediated tumor microenvironment at the protein level^63^. The highly immunogenic property of HPV16 virus, the dominant HPV subtype affecting HNSC patients, can help to explain the increased immune response in HPV-positive HNSC samples (Supplementary Fig. 15a). There is no significant difference in terms of mutational burden in HPV-positive and virus-negative samples (Supplementary Fig. 15b). However, why different patients have different responses to different viral presentation requires more detailed work in the future. In addition, though the current work does not identify clear mechanisms by which virus infection affects *PD-L1* and *PD-L2* expression, it nonetheless suggests that viruses may aid tumors in evading the PD-1 immune checkpoint pathway across multiple cancer types. Our analysis of elevated expression in both *PD-1*, *CTLA-4*, *Tim-3*, *LAG3*, and *4-1BB* checkpoint genes and immune response in virus-positive tumors may contribute to therapy selection in these patients.

## Methods

### Virus integration

We discovered viruses in the tumor samples by using the VirusScan pipeline^30^, which is available from Github^64^. For the identification of virus integration sites in human genome, we first used BWA^65^ to align RNA-Seq data to the human plus viral reference. From the re-aligned bam file, we extracted the discordant read pairs, where one read of a read pair maps to human, the other to virus. Pindel^66^ was used to identify exact breakpoints for all samples with ten or more human-virus discordant reads. The breakpoints in Fig. 1 were visualized by using BreakPointSurveyor^67^.

### Statistical analysis

Survival analysis was implemented by using R package survival. We used the Student’s *t*-test to extract differentially expressed genes in virus-positive and negative samples, using FDR = 0.05 as the cut-off. The FDR value was obtained by p.adjust with Benjamini and Hochberg correction from R package. The heatmap figure was generated by using *Heatmap.3*R package with default parameters.

### Expression outlier analysis

To investigate if genes with virus integrations are expression outliers, we used the Tukey’s standard formula to quantify the outlier score:

Outlier score = (*x* − Q3)/IQR for upper tail and (*x* − Q1)/IQR for low tail,

where IQR is the interquartile range, Q1 and Q3 are the first and third quartiles, respectively and *x* is the RSEM value in a log2 scale. In the current study, genes with an outlier score greater than 1.0 or less than −1.0 are considered to be expression outliers.

### Neoantigen prediction

Different lengths of epitopes (8mer, 9mer, 10mer, and 11mer) are constructed from HPV16 protein sequences. We use NetMHC3pan^68^ to predict the binding affinity between epitopes and MHC based on the HLA type in each tumor. The HLA type was adopted from ref. ^69^. Epitopes with binding affinity ≤500 nM which are also not present in Ensembl 70.37 database are extracted for the following neoantigen analysis.

### Reporting Summary

Further information on experimental design is available in the Nature Research Reporting Summary linked to this article.

## Supplementary information

Supplementary Information

Reporting Summary

Supplementary Data 1

Description of Additional Supplementary Files

## Data Availability

We collected gene expression (RSEM), and clinical data from Broad firehose^70^ across six cancer types including cervical squamous cell carcinoma and endocervical adenocarcinoma (CESC), colon adenocarcinoma and rectal adenocarcinoma (COADREAD), esophageal cancer (ESCA), head/neck squamous cell carcinoma (HNSC), stomach adenocarcinomas (STAD), and liver hepatocellular carcinoma (LIHC) from The Cancer Genome Atlas (TCGA). The aligned TCGA RNA-Seq bams included in this study can be downloaded from the NCI’s Genomic Data Commons (GDC). The source gene expression data are available in the Supplementary Data 1.
