# Supplementary material for: Dynamic host immune response in virus-associated cancers
Source: Commun Biol. 2019 Mar 22;2:109. doi: 10.1038/s42003-019-0352-3 (PMC6430765; doi:10.1038/s42003-019-0352-3)
Supplement: Supplementary file 1 — Supplementary Information [file 42003_2019_352_MOESM1_ESM.pdf]

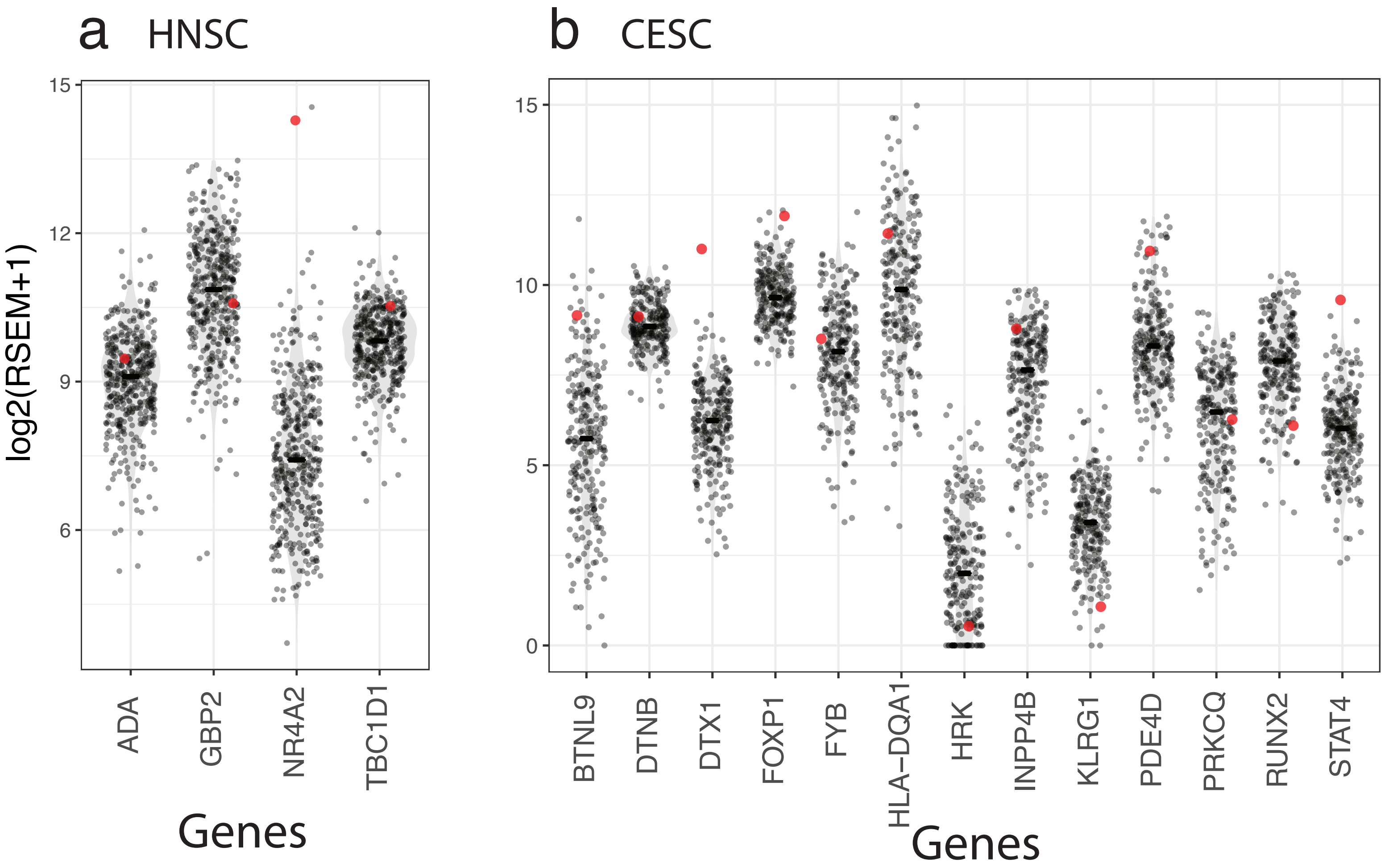

Supplementary Figure 1: Expression of T-Cell and B-Cell genes with and without HPV integrations in (a) HNSC and (b) CESC. Dots marked by Red indicate samples HPV integrations.

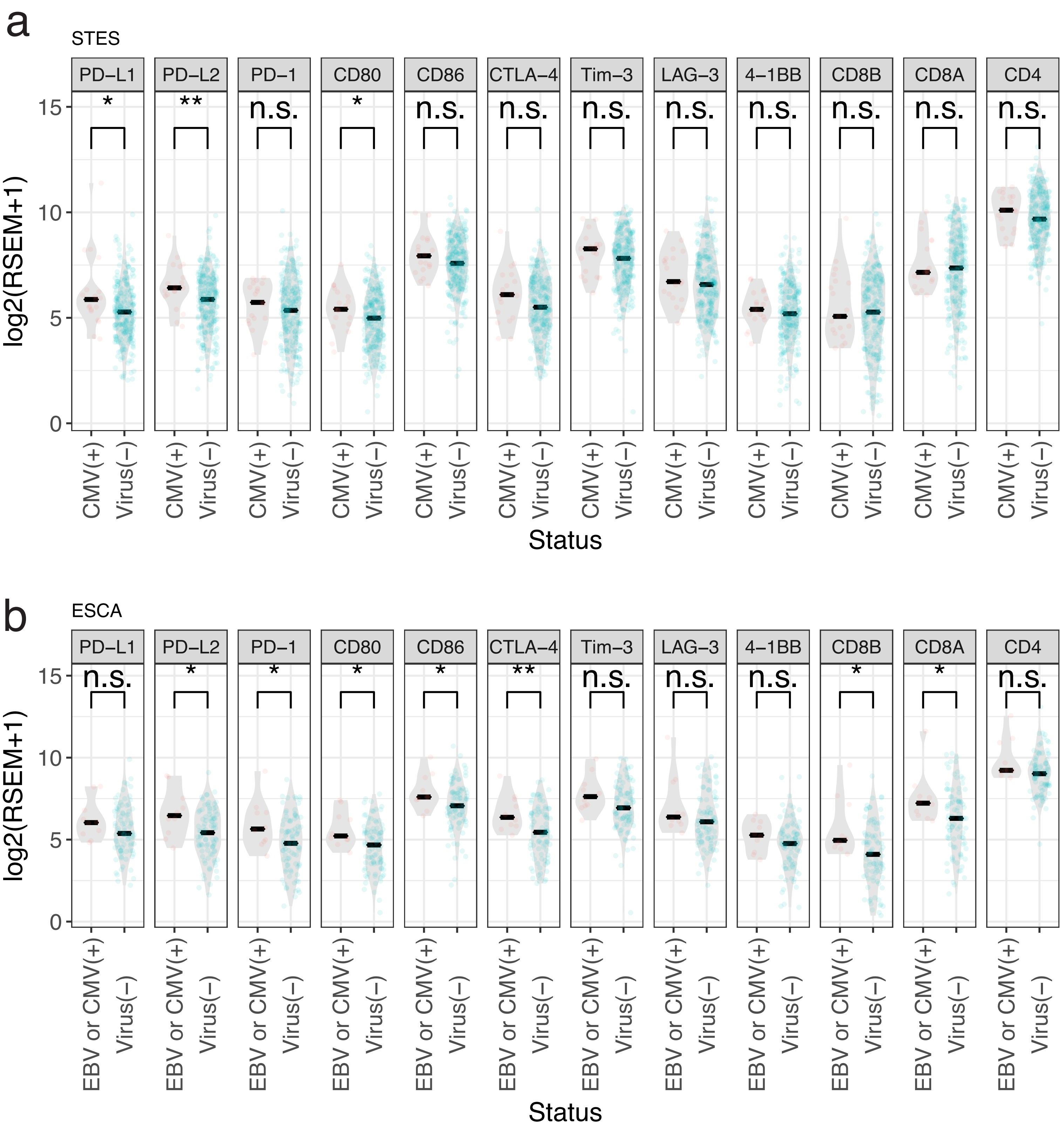

Supplementary Figure 2: The comparison of *PD-L1*, *PD-L2*, *PD-1*, *CD80*, *CD86*, *CTLA-4*, *TIM-3*, *LAG-3*, *4-1BB*, *CD8B, CD8A*, and *CD4* expressions in (a) CMV-positive and negative STES tumors and (b) EBV or CMV positive and negative ESCA tumors.

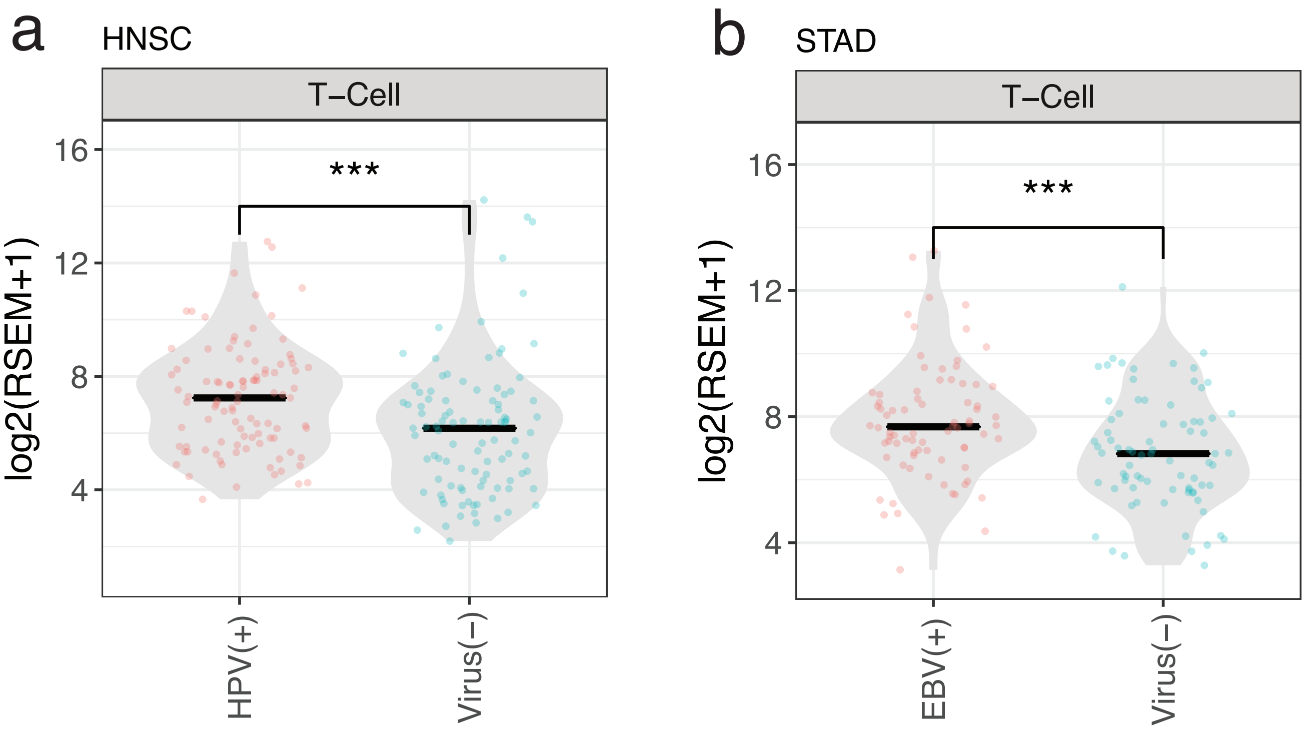

Supplementary Figure 3: The comparison of medians of differentiallly expressed T-Cell genes in Fig. 3a and b between (a) HPV+ and virus- HNSC tumors, and (b) EBV+ and virus- STAD tumors.

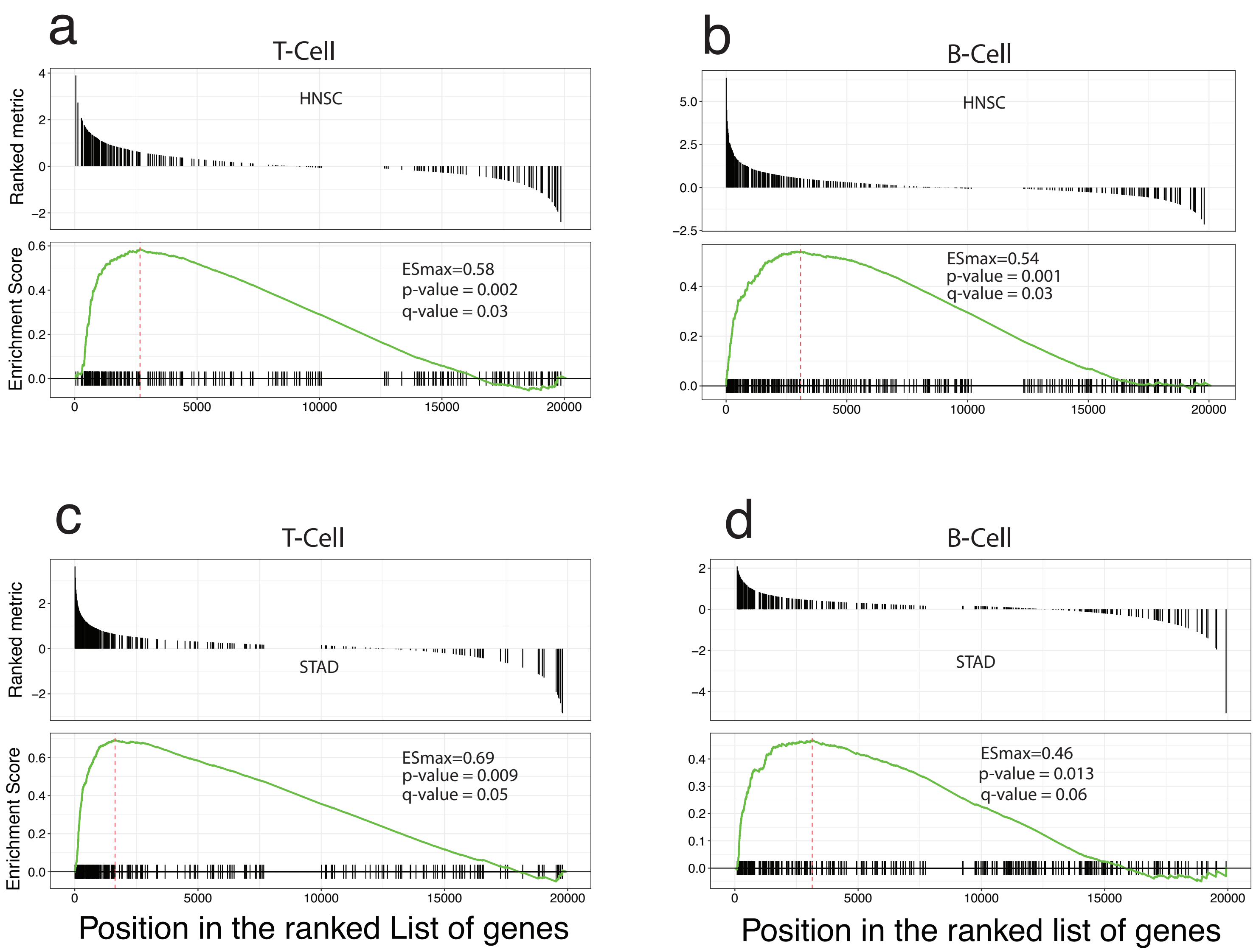

Supplementary Figure 4: GSEA analysis of T-Cell and B-Cell gene sets (a) and (b) in HPV-positive HNSC tumors against virus-negative tumors, (c) and (d) in EBV-positive STAD tumors against virus-negative tumors. In the analysis, we use the difference of medians of the expressions of T-Cell and B-Cell genes (in log2 scale) between HPV-positive or EBV-positive samples and virus-negative samples as ranked metric. We found an enrichment of T-Cell and B-Cell gene sets in HPV-positive and EBV-positive samples with significant p-value and q-value below or close 0.05.

GO annotation of differentially expressed T−Cell genes

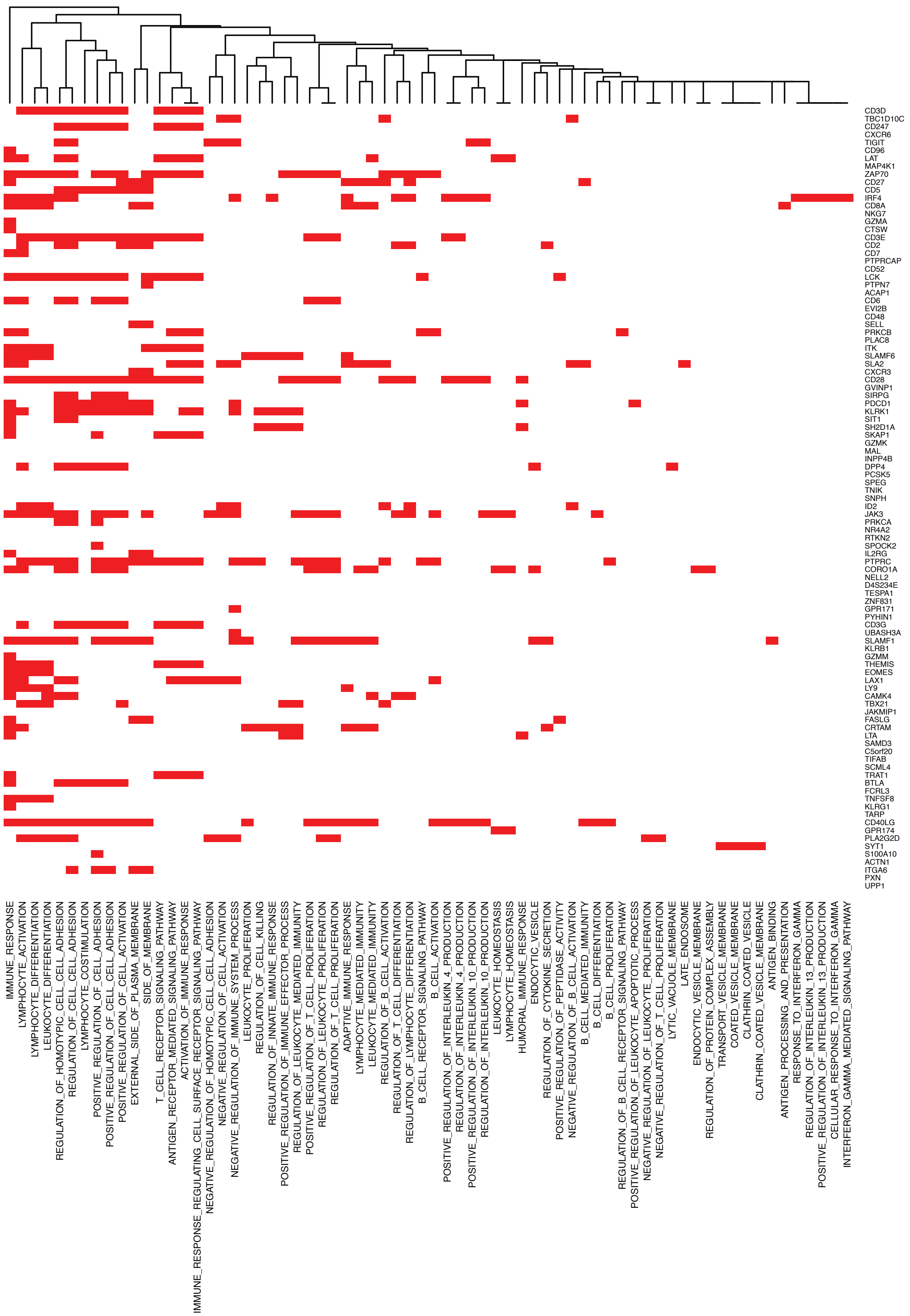

Supplementary Figure 5: The gene oncology (GO) annotation of differentially expressed T-Cell genes in HPV-positive and negative tumors of Fig. 3.

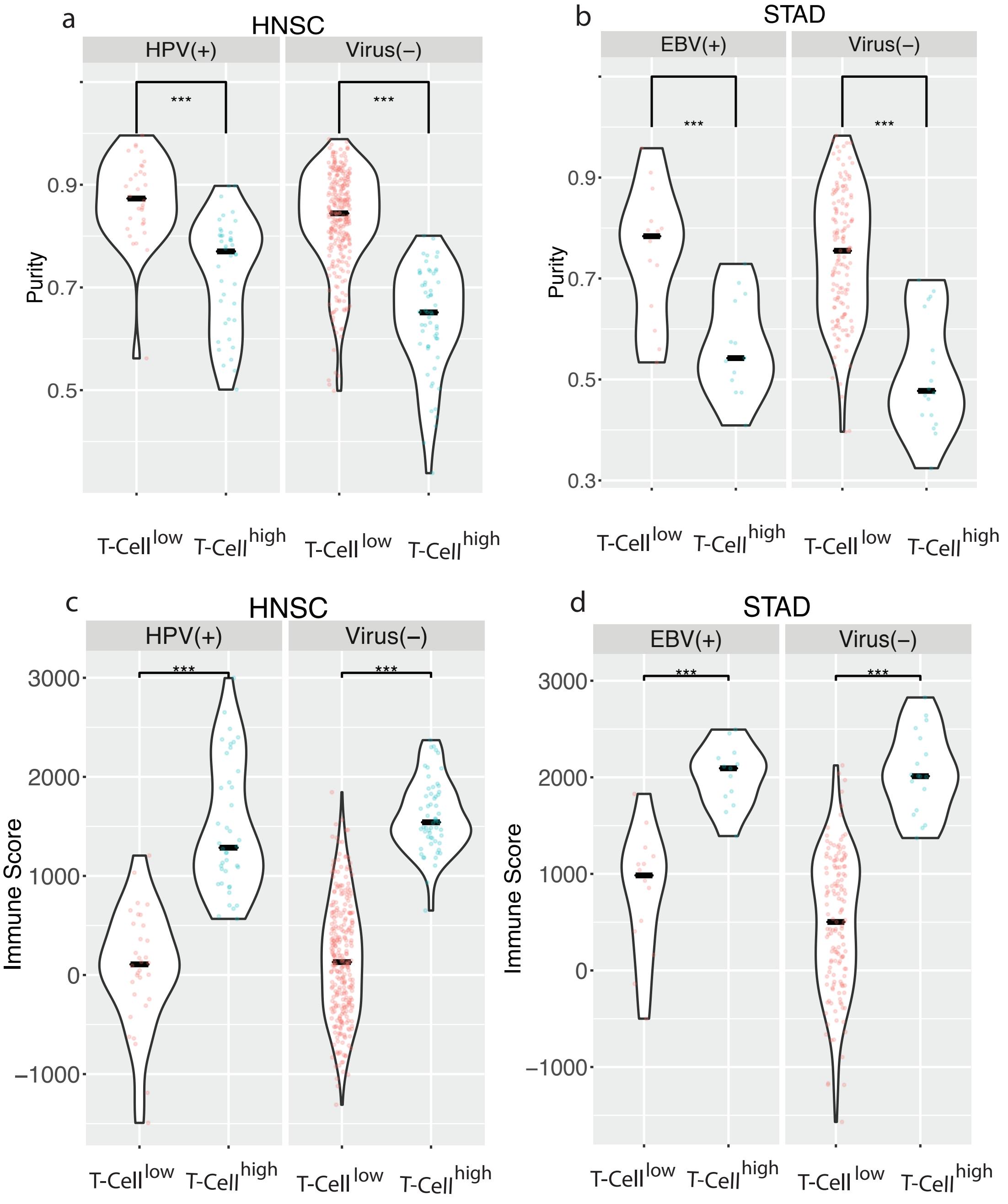

Supplementary Figure 6: The comparison of tumor purity and immune score between T-Cell high and T-Cell low samples.

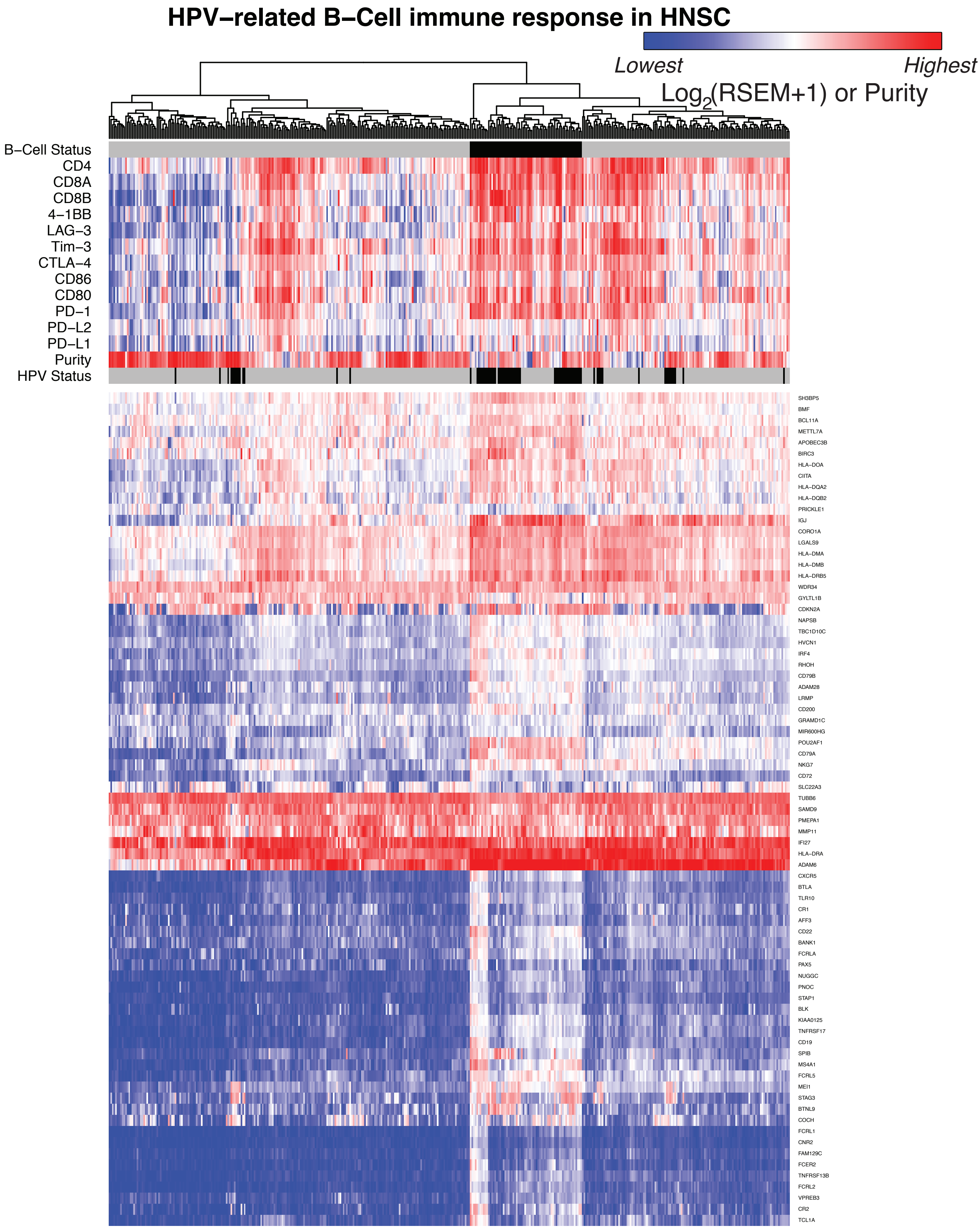

Supplementary Figure 7: The supervised cluster of B-Cell significant genes for HPV-positive and virus-negative HNSC samples.

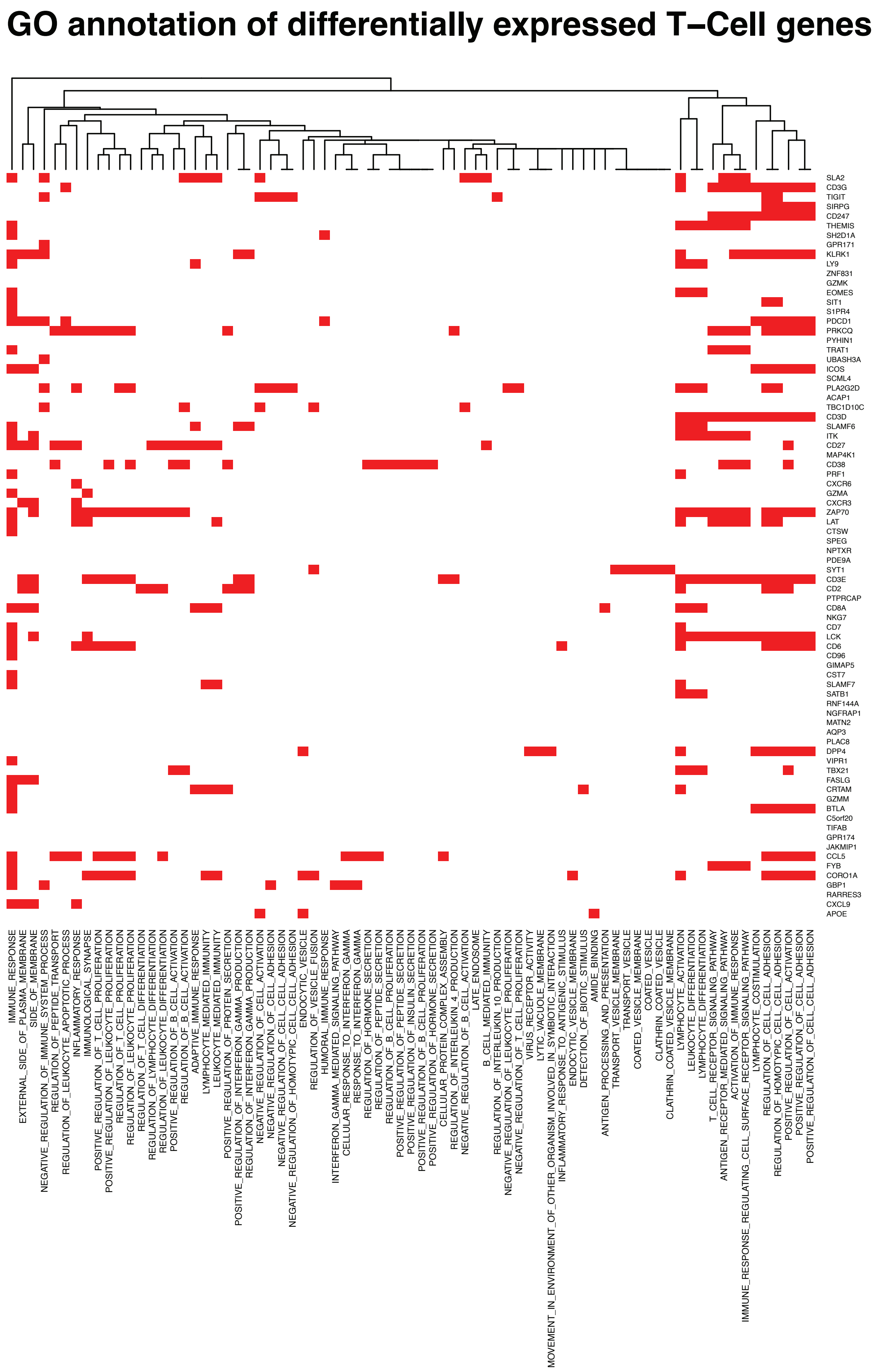

Supplementary Figuire 8: The gene oncology (GO) annotation of differentially expressed T-Cell genes between EBV-positive and virus-negative STAD tumors of Fig. 4.

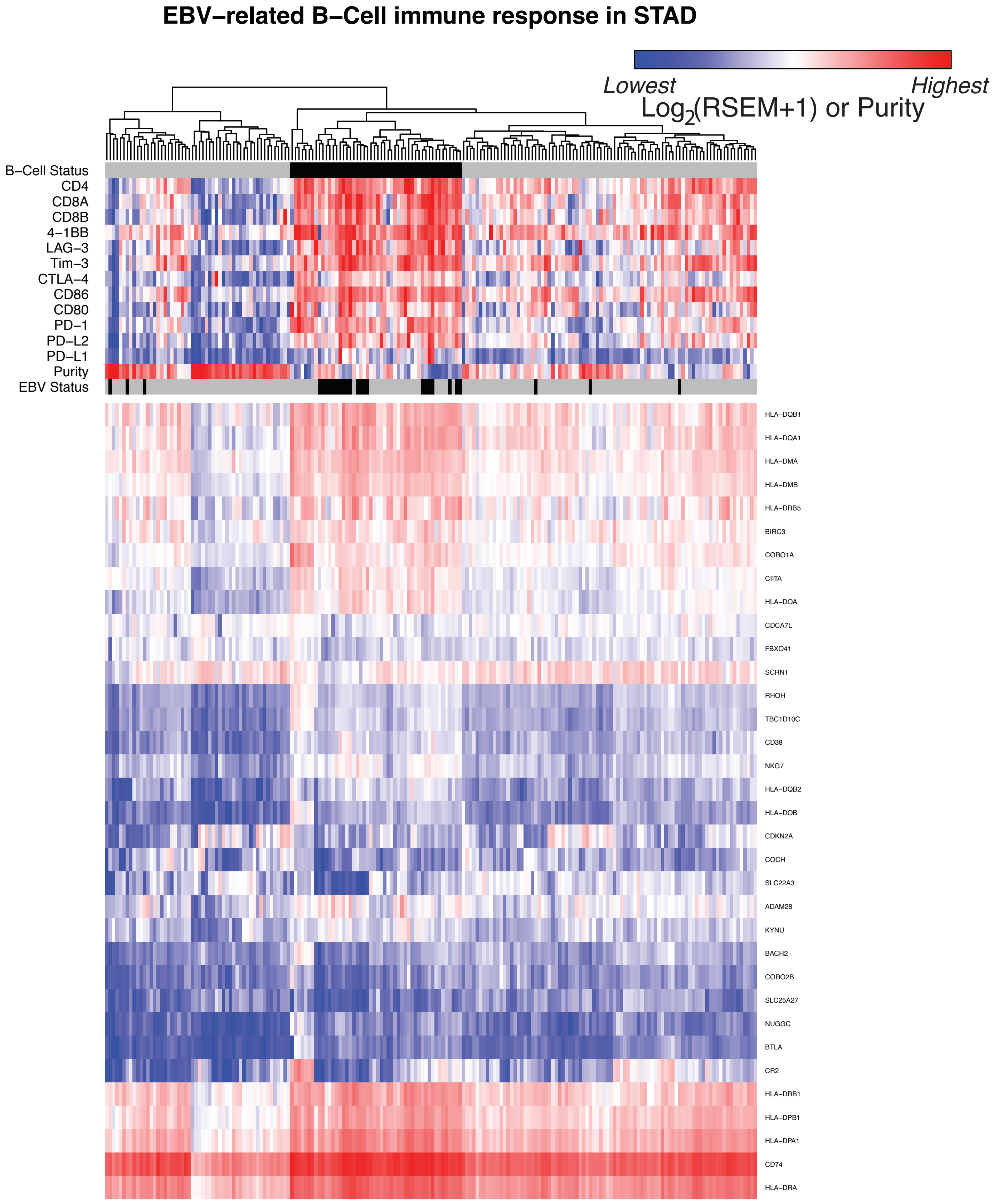

Supplementary Figure 9: The supervised cluster of B-Cell significant genes for EBV-positive and virus-negative STAD tumors.

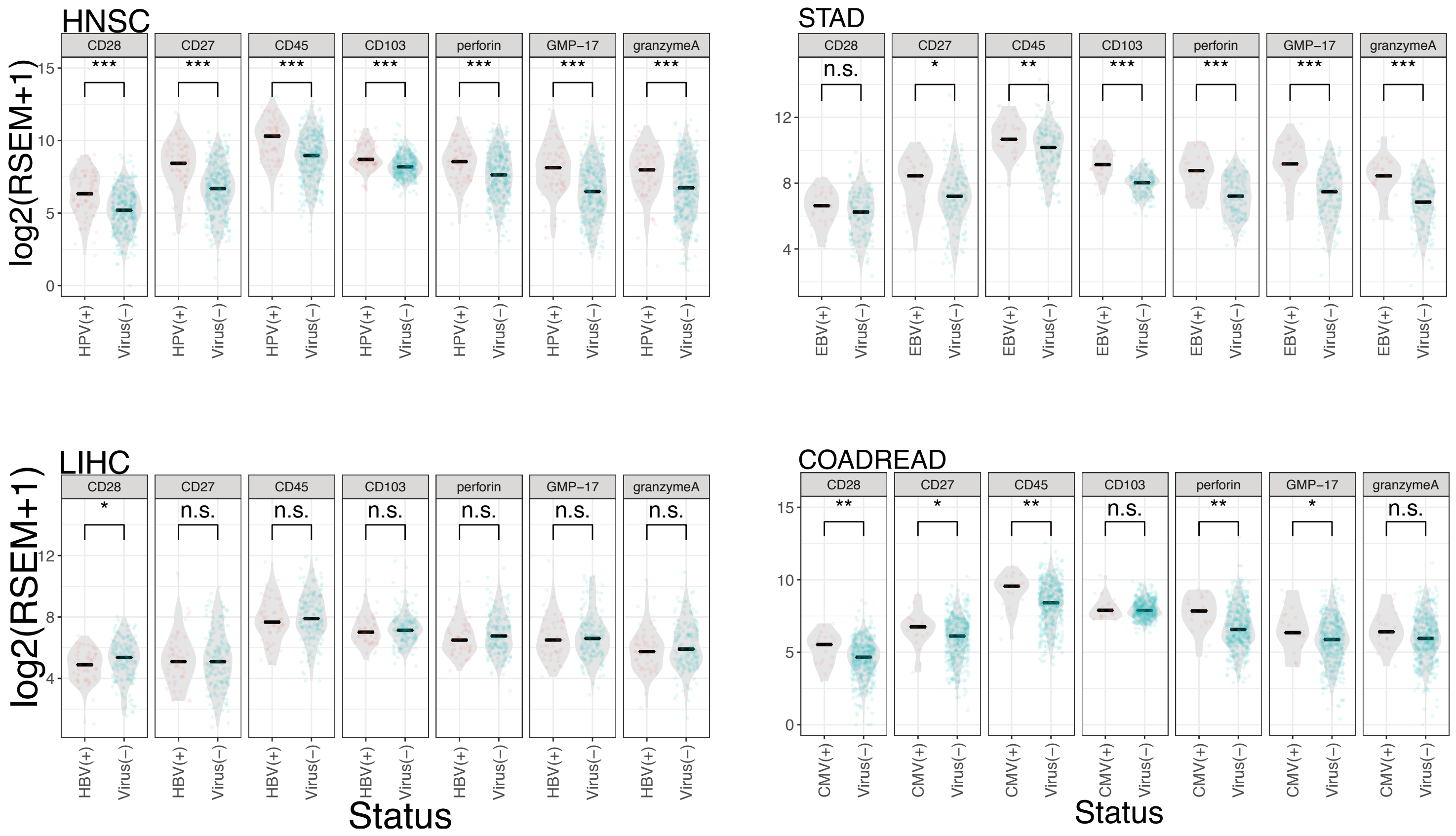

Supplementary Figure 10: The comparison of expressions of *CD28*, *CD27*, *CD45*, *CD103*, *perforin*, *GMP-17*, and *granzymeA* between different virus-positive and negative tumors.

HPV−related T−Cell immune response in CESC

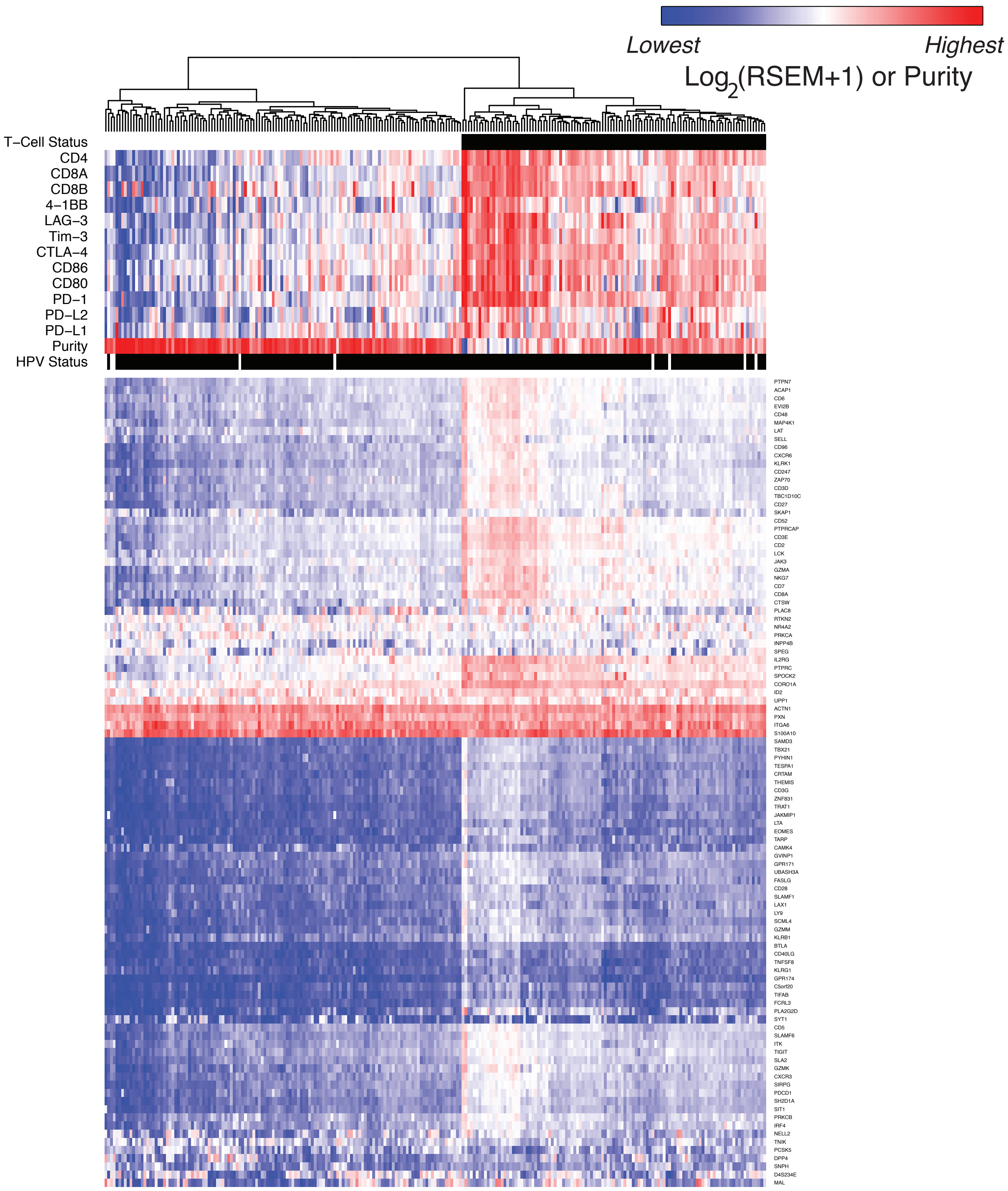

Supplementary Figure 11: The clustering of T-Cell high and T-Cell low tumors in CESC based on the T-cell signature found in HPV-positive HNSC tumors.

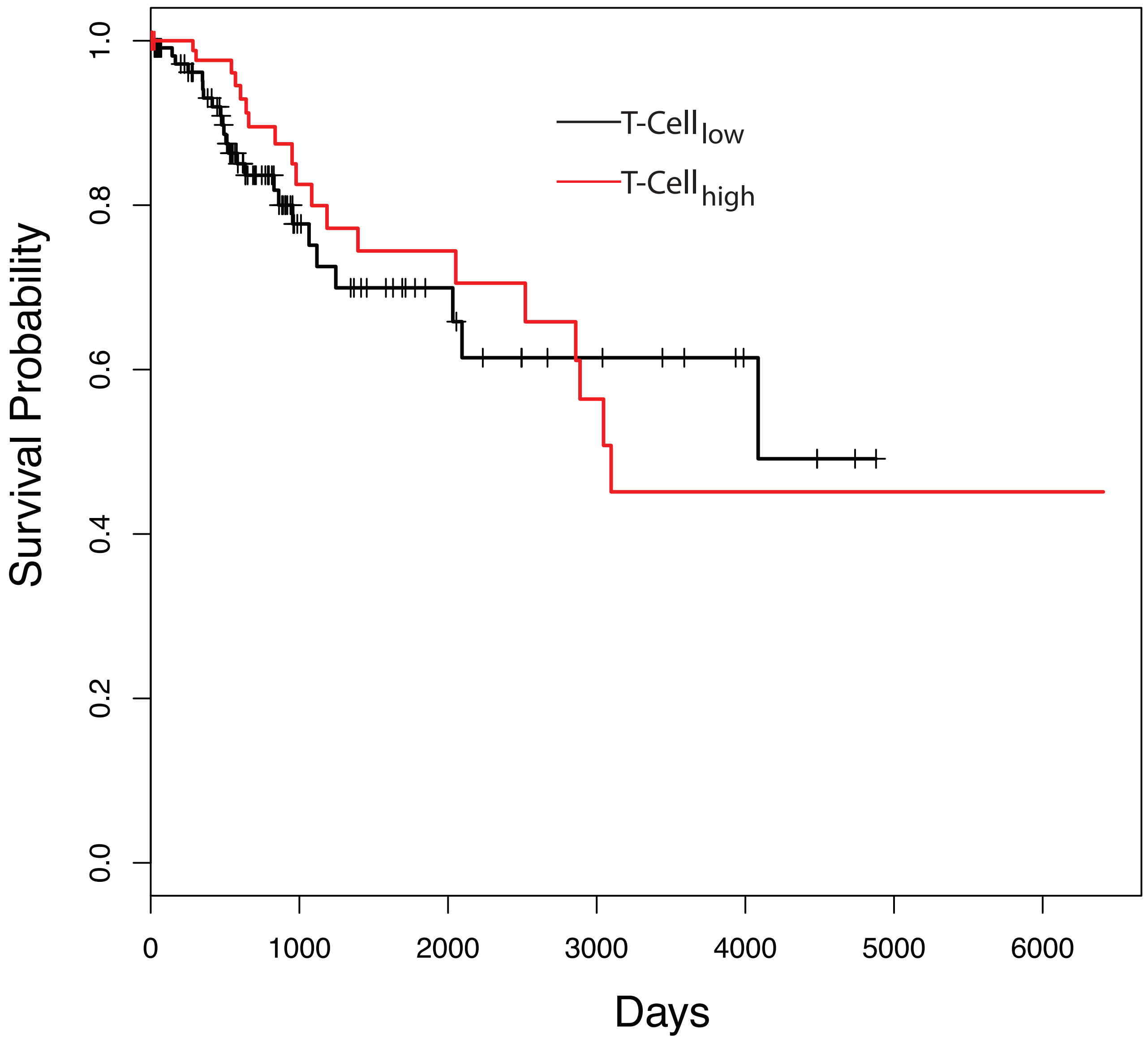

Supplementary Figure 12: The comparison of overall survival in T-Cell high and T-Cell low tumors in HPV-positive CESC tumors.

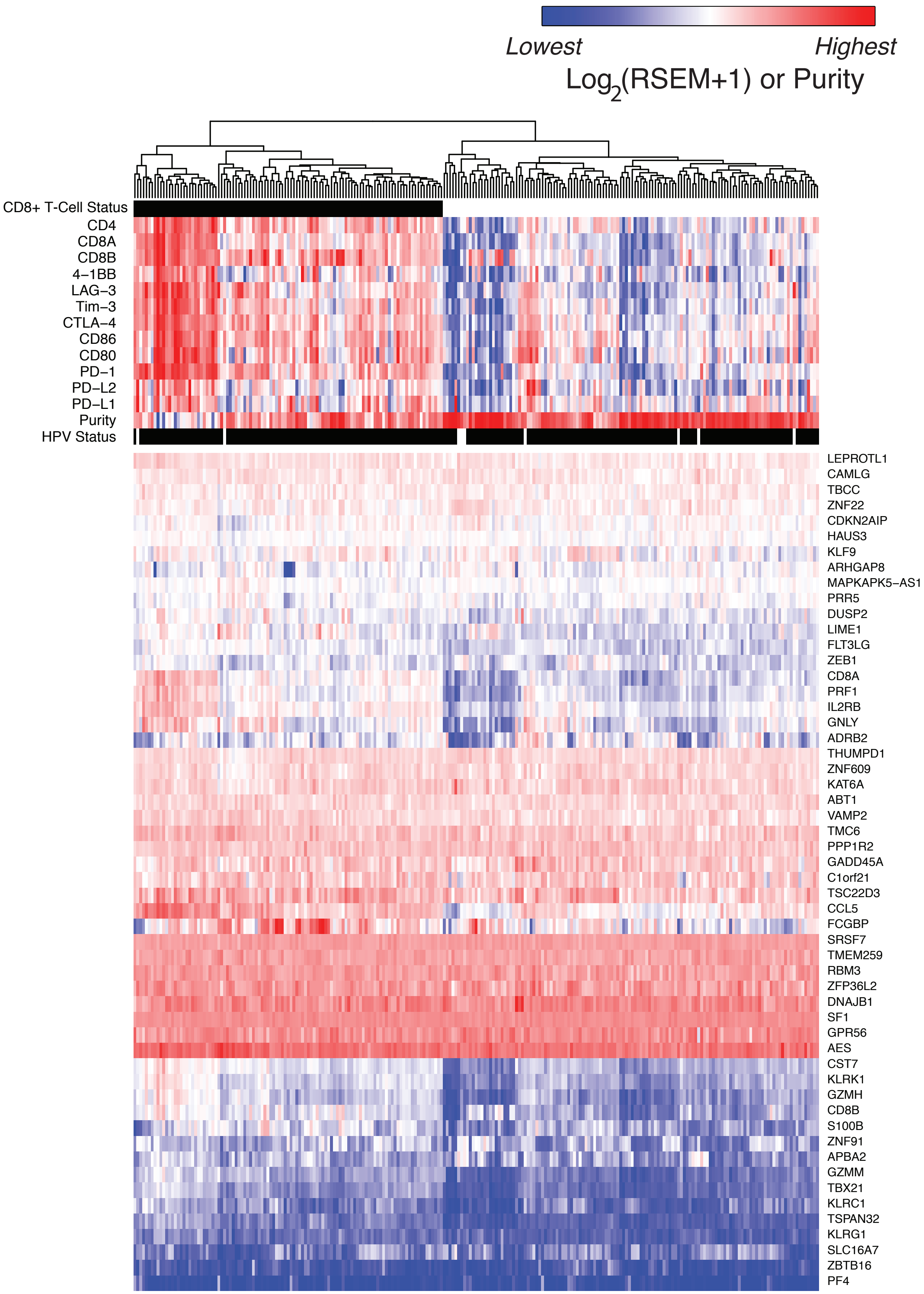

Supplementary Figure 13: Unsupervised clustering of CESC tumors based on CD8+ T-Cell genes.

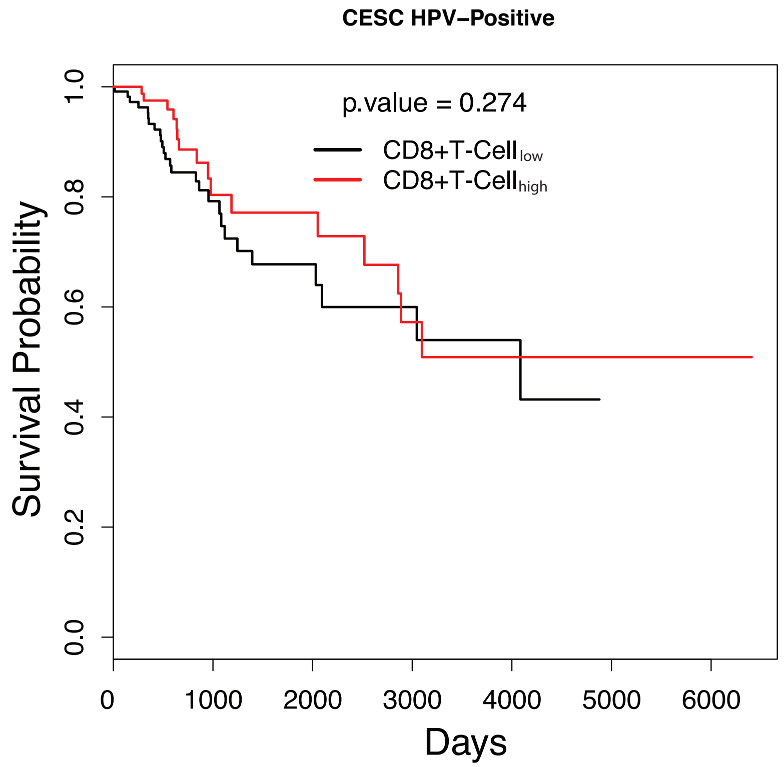

	Tumor free	With Tumor
CD8+T-Cell$_{low}$	75	35
CD8+T-Cell$_{high}$	77	15

p-value = 0.01 from Fisher's Exact Test

Supplementary Figure 14: The comparison of overall survival in CD8+T-Cell high and CD8+T-Cell low samples in HPV-positive CESC samples. In the right panel, we showed the number of samples with “Tumor free” and “With Tumor”. Tumors with CD8+T-Cell high status have a high chance of “Tumor free” status in the following check-ups (p-value = 0.01).

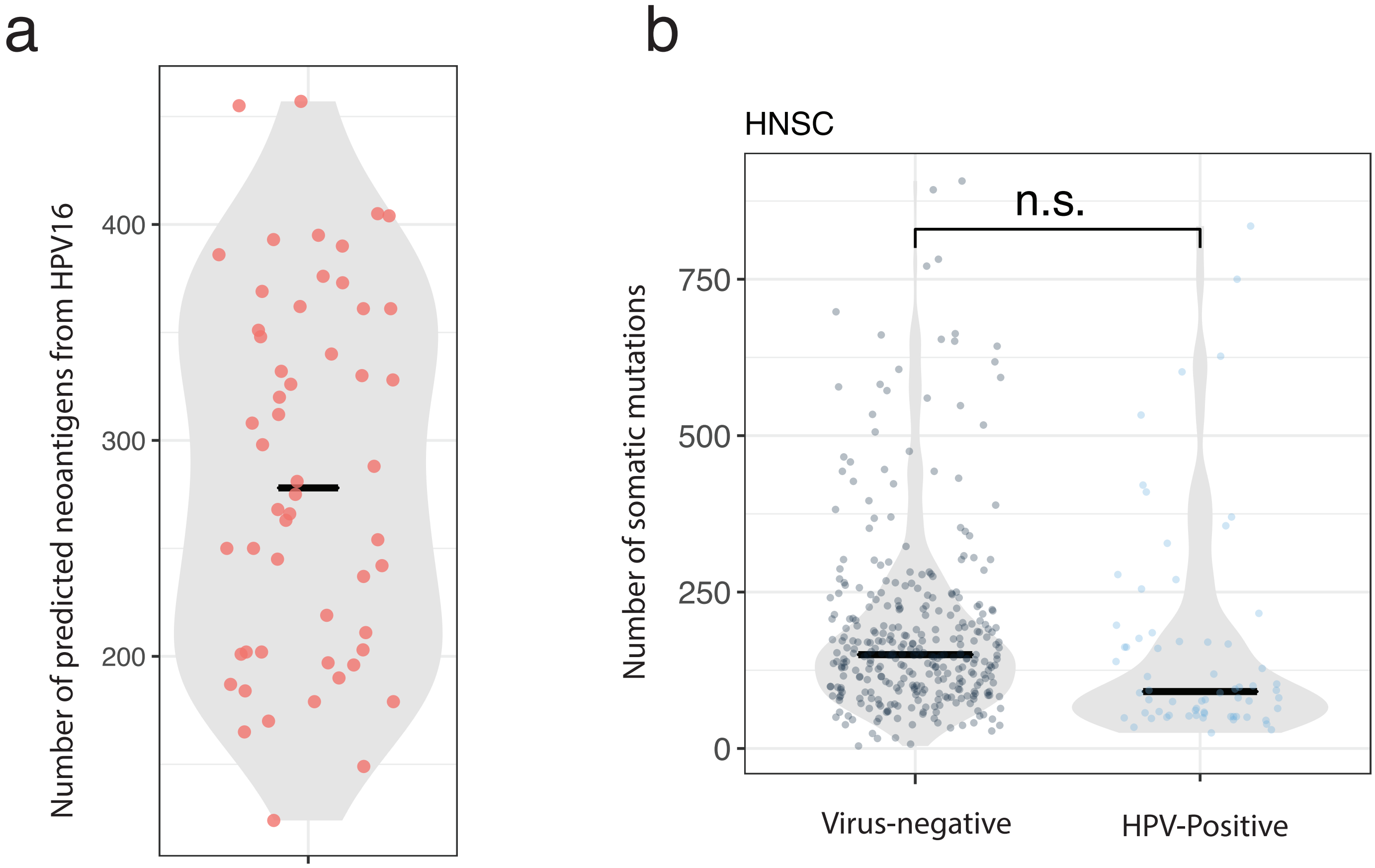

Supplementary Figure 15: (a) Number of predicted neoantigens from HPV16 across different HPV16-postive HNSC tumors. HPV16 is the dominant HPV subtype which can be found in 84% of HPV-positive HNSC tumors. (b) The comparison of mutational burden in HPV-positive and virus-negative tumors.

Supplementary Table 1: The association between virus status, sex and ethnicity.

HNSC	MALE	FEMALE	p-value
HPV+	66	6	0.0002
Virus negative	244	97	

STAD	MALE	FEMALE	p-value
EBV+	23	4	0.005
Virus negative	91	72	

LIHC	MALE	FEMALE	p-value
HBV+	41	8	0.0034
Virus negative	104	66	

COADREAD	MALE	FEMALE	p-value
CMV+	9	3	0.24
Virus negative	162	128	

HNSC	CAUCASIAN	BLACK or AFRICAN AMERICAN	p-value
HPV+	69	3	0.32
Virus negative	295	27	

STAD	CAUCASIAN	ASIAN	p-value
EBV+	16	9	0.82
Virus negative	93	48	

LIHC	CAUCASIAN	ASIAN	p-value
HBV+	2	42	0.0001
Virus negative	142	16	

COADREAD	CAUCASIAN	BLACK or AFRICAN AMERICAN	p-value
CMV+	11	0	0.22
Virus negative	216	41	

Supplementary Table 2: The T-Cell and B-Cell gene list

T-Cell: AAK1 ACAP1 ACTN1 ACVR2B ADA AMICA1 ANXA1 APBA2
SPEG APOE AQP3 ARL4C ATP1A1 BAG3 BCL11B BIN2 BTLA
BUB1B C16orf54 RNF213 C20orf112 C5orf20 CAMK4 CCL5
CCND2 CCR2 CD2 CD247 CD27 CD28 CD38 CD3D CD3E
CD3G CD40LG CD48 CD5 CD52 CD53 CD6 CD7 CD8A
CD96 CDC14A CDC25B CDR2 CISH CORO1A CRTAM CST7 CTLA4
CTSW CXCL9 CXCR3 CXCR6 D4S234E NBPF14 DNAJB1 DNASE1L3
DOCK9 DPP4 ARID3A NAP1L5 DUSP16 DUSP2 LPAR2 FAM102A EOMES
LIMA1 TMC6 EVI2B FASLG FBLN5 FCRL3 FHIT MAP7D1 DNAJC17
FAM134B MORC2-AS1 FLT3LG AKTIP FYB FYN GABARAPL1
GALT GATA3 GBP1 GBP2 GFI1 GIMAP2 GIMAP4 GIMAP5 GPR171
GPR174 GPSM3 GVINP1 GZMA GZMK GZMM HOXB2 HSPA1L ICOS
ID2 IFITM1 IL10RA IL18R1 IL2RG IL6R IL6ST IL7R INPP4A
INPP4B IRF4 ITGA6 ITK ITM2A ITPKB JAK3 ARHGAP25
TESPA1 KLRB1 KLRG1 KLRK1 LAPTM5 LAT LAX1 LCK LCP2
LCP:7125 LDHA LDHB LEF1 LEPROTL1 LINC00426
LOC112868 LOC283666 LOC340061 LPIN2 LRIG1 LTA
LTBP4 LY9 LYAR MAL MAN1C1 MAP4K1 MAPKAPK5 JAKMIP1
MAST4 MATN2 MEN1 PIK3IP1 SLFN5 COA5 MLLT3 MPP7 MYBL1
NCALD NELL2 NGFRAP1 IL32 NKG7 NPDC1 NPTXR NR4A2 OPTN
LPAR6 PAG1 PCSK5 PCYT2 PDCD1 PDE4D PDE9A PIK3R1 PIM1
PKM PLA2G2D PLAC8 RTKN2 PLXDC1 PRF1 PRKCA PRKCB PRKCI
PRKCQ PSTPIP1 PTGER2 PTPN7 PTPRC PTPRCAP PXN PYHIN1 RAB43
RARRES3 RASGRP1 RBMS1 RGS10 GIPC1 RNF144A RORA RUNX2 S100A10
S100A8 S1PR4 SAMD3 SAMSN1 SATB1 SCML4 SELL SELPLG SEMA4D
TSEN54 SH2D1A SHFM1 SIRPG SIT1 SKAP1 SLA2 SLAMF1 SLAMF6
SLAMF7 SLCO3A1 SLC35D2 SLC39A8 SNPH SOCS3 SORL1 SP140 SPOCK2
STAT4 SYNE2 SYT1 TACC3 TARP TBC1D10C TBX21 TCF7
TRAT1 OLAH THEMIS TIAM1 TIFAB TIGIT TSPAN14 TNFAIP3
TNFRSF25 TNFSF8 TNIK TOB1 TRA TRB TRBC1 TRERF1
CEP41 TXK UBASH3A UPP1 VIPR1 LINS WNT10B WWP1 ZAP70
ZNF831

B-Cell: ABCA1 ABCB4 ACTA2 ADAM19 ADAM28 ADAM6 ADK
ADRBK2 AIM2 ALOX5 AMFR ANXA4 ATG4A APOBEC3B
ARHGAP10 ARHGEF3 ATP5B ATP6V0A1 BACE2 BACH2 BANK1
BCL11A BCL7A FAM129C BIRC3 BLK BLNK CXCR5 BMF BMS1P20
BRD4 STAP1 BSG BTK BTNL9 C11orf24 KIAA0226L
MGME1 GUCD1 CCDC50 CCNG2 CCR9 CD19 CD1C CD1D MS4A1
CD200 CD22 CD24 CD72 CD74 CD79A CD79B CD83 CD86
CDKN2A CEBPB CHD7 CHERP PLEKHO1 CLIC4 CNR2 COCH COL14A1
COPS3 CORO1C CORO2B CR1 CR2 CTSH CTSZ CYB561D2
CYB561A3 CYBB CYSLTR1 DAPP1 DDR1 METTL7A GRAMD1C DMXL1
DTNB DTX1 E2F5 EBF1 UBR5 EGR1 EPB41L2 EPHX1 DNAJC10
EVI5L F5 KIAA0125 FBXO10 FBXO41 FCER2 FCGR2B FCRL1
FCRL2 FCRL5 FCRLA FGD2 FLII ZNF532 CCDC25 VPS53 SNX29

TCTN1 RALGPS2 FOXP1 FUBP1 FZD5 RAP1GAP2 GCNT1
MIR600HG GLDC GM2A GNA12 GNG7 GSTZ1 GYLTL1B HECW2
HERPUD1 HHEX HIST1H2BK HLA-DMA HLA-DMB HLA-DOA HLA-DOB HLA-DPA1 HLA-DPB1 HLA-DQA1 HLA-DQA2 HLA-DQB1
HLA-DQB2 HLA-DRA HLA-DRB1 HLA-DRB5 HLA-DRB6
HRK HSPA5 HSPA6 SSU72 PPAPDC1B IFI27 IFIT3 IFNGR2
IGH IGHA1 IGHD IGHG1 IGHG3 IGHM IGJ IGKC IGKV1-5
IGKV1D-13 IGL IGLC2 IGLJ3 IGLL1 IGLL3P IL4R INPPL1
IRF8 ITGB1 ITPR1 KDM4B JUP FIG4 DENND4B SEL1L3 KAZN
RALGAPB KLF1 KLHL14 KYNU AFF3 LCP:21 LGALS9 LHFPL2
LOC201895 LOC283663 LOC339562 LOC348938
LOC388078 LOC391427 LOC440871 LOC51760 GSAP
LOC57228 LOC91316 LOC92497 LRMP CD180 LY86
LYN MAP3K8 1-Mar MARCKS MEF2C MEI1 HVCN1 DENND5B
MGC27165 TSPAN33 DRAM2 CIITA MICAL3 RCSD1 MMP11 MOB3B
MRPL49 MTPN MTSS1 MYBL2 MYO1E NAP1L NAPSB NCF1 NCF4
TSPAN9 NFKBIE NUGGC NUP88 ODC1 OSBPL10 PACAP PALM2-AKAP2
PARP14 PAX5 PCCA PEA15 PHF16 PIK3AP1 PIK3C2B PLCG2 PLEKHF2
PMAIP1 PNOC POLD4 POU2AF1 POU2F2 PPP3CA PRCP PRICKLE1
PRKCE CYTH1 PSEN2 WDR83OS QRSL1 RAB30 CDCA7L RFX5 RGS13
RHOBTB2 RHOH RIPK2 RNASE6 RNF141 USP6NL RRAS2 SAMD9 TSPAN31
SAV1 SKAP2 SCN3A SCRN1 SEMA4B SETBP1 SH3BP5 SHMT2 SIDT2
SLC15A2 SLC22A3 SLC2A1 SLC2A5 SLC7A7 SMAD3 SMC6 SNX10 SNX2
SPI1 SPIB SRGAP2 SSPN ST14 STAG3 STAT6 STRBP STX7
SWAP70 SYK SYNGR2 SYPL1 TBC1D1 TCF4 TCL1A TEAD2 TNS3
TFEB TLR10 TLR7 TSPAN3 TMED8 PMEPA1 TNFRSF13B
TNFRSF17 TNFRSF18 TPD52 TRIM26 TRIM56 TRIO TTC7A
TUBB6 UBE2J1 SLC25A27 SUN2 HUWE1 UROS UVRAG VPREB3
VPS28 WIPF1 WDR11 WDR34 WEE1 XYLT1 ZCCHC7 ZNF154 ZNF207
