# Supplementary material for: Dynamic host immune response in virus-associated cancers
Source: Commun Biol. 2019 Mar 22;2:109. doi: 10.1038/s42003-019-0352-3 (PMC6430765; doi:10.1038/s42003-019-0352-3)
Supplement: Supplementary file 2 — Reporting Summary [file 42003_2019_352_MOESM2_ESM.pdf]

nature research

Corresponding author(s): Li Ding

Last updated by author(s): Jan 16, 2019

Reporting Summary

Nature Research wishes to improve the reproducibility of the work that we publish. This form provides structure for consistency and transparency in reporting. For further information on Nature Research policies, see Authors & Referees and the Editorial Policy Checklist.

Statistics

For all statistical analyses, confirm that the following items are present in the figure legend, table legend, main text, or Methods section.

n/a	Confirmed	
☐	☒	The exact sample size (n) for each experimental group/condition, given as a discrete number and unit of measurement
☐	☒	A statement on whether measurements were taken from distinct samples or whether the same sample was measured repeatedly
☐	☒	The statistical test(s) used AND whether they are one- or two-sided<br>*Only common tests should be described solely by name; describe more complex techniques in the Methods section.*
☒	☐	A description of all covariates tested
☐	☒	A description of any assumptions or corrections, such as tests of normality and adjustment for multiple comparisons
☒	☐	A full description of the statistical parameters including central tendency (e.g. means) or other basic estimates (e.g. regression coefficient) AND variation (e.g. standard deviation) or associated estimates of uncertainty (e.g. confidence intervals)
☐	☒	For null hypothesis testing, the test statistic (e.g. F, t, r) with confidence intervals, effect sizes, degrees of freedom and P value noted<br>*Give P values as exact values whenever suitable.*
☒	☐	For Bayesian analysis, information on the choice of priors and Markov chain Monte Carlo settings
☒	☐	For hierarchical and complex designs, identification of the appropriate level for tests and full reporting of outcomes
☒	☐	Estimates of effect sizes (e.g. Cohen's d, Pearson's r), indicating how they were calculated

Our web collection on statistics for biologists contains articles on many of the points above.

Software and code

Policy information about availability of computer code

Data collection	n/a
Data analysis	We use previously published tools and standard R packages for the analysis. We mentioned them in the methods.

For manuscripts utilizing custom algorithms or software that are central to the research but not yet described in published literature, software must be made available to editors/reviewers. We strongly encourage code deposition in a community repository (e.g. GitHub). See the Nature Research guidelines for submitting code & software for further information.

Data

Policy information about availability of data

All manuscripts must include a data availability statement. This statement should provide the following information, where applicable:

- Accession codes, unique identifiers, or web links for publicly available datasets
- A list of figures that have associated raw data
- A description of any restrictions on data availability

We collected gene expression (RSEM), and clinical data from Broad firehose (http://gdac.broadinstitute.org/runs/stddata__2016_01_28/) across six cancer types including cervical squamous cell carcinoma and endocervical adenocarcinoma (CESC), colon adenocarcinoma and rectal adenocarcinomas (COADREAD), esophageal cancer (ESCA), head/neck squamous cell carcinoma (HNSC), stomach adenocarcinomas (STAD), and liver hepatocellular carcinoma (LIHC from The Cancer Genome Atlas (TCGA). The aligned TCGA RNA-Seq bams included in this study can be downloaded from the NCI's Genomic Data Commons (GDC).

Field-specific reporting

Please select the one below that is the best fit for your research. If you are not sure, read the appropriate sections before making your selection.

☒ Life sciences ☐ Behavioural & social sciences ☐ Ecological, evolutionary & environmental sciences

For a reference copy of the document with all sections, see nature.com/documents/nr-reporting-summary-flat.pdf

Life sciences study design

All studies must disclose on these points even when the disclosure is negative.

Sample size	2,009 tumor data from The Cancer Genome Atlas (TCGA)
Data exclusions	We include tumor data with a clear virus-negative and positive status based on the previous publication (Cao et al., Scientific Reports, 2016), and exclude tumor data without a clear virus status.
Replication	We use all virus-positive and negative tumors in each cancer type to maximize the statistical power, and did the confirmation of the findings of the increase of PD-L1 and PD-L2 expression found in CMV-positive COADRAD by using CMV-positive STES tumors.
Randomization	Tumors are separated into case and control groups purely based on the virus status.
Blinding	The tumor data are provided by TCGA, and we are blind to the sample selection.

Reporting for specific materials, systems and methods

We require information from authors about some types of materials, experimental systems and methods used in many studies. Here, indicate whether each material, system or method listed is relevant to your study. If you are not sure if a list item applies to your research, read the appropriate section before selecting a response.

Materials & experimental systems

n/a	Involved in the study	
☒	☐	Antibodies
☒	☐	Eukaryotic cell lines
☒	☐	Palaeontology
☒	☐	Animals and other organisms
☒	☐	Human research participants
☒	☐	Clinical data

Methods

n/a	Involved in the study	
☒	☐	ChIP-seq
☒	☐	Flow cytometry
☒	☐	MRI-based neuroimaging
