# Supplementary material for: Dynamic host immune response in virus-associated cancers
Source: Commun Biol. 2019 Mar 22;2:109. doi: 10.1038/s42003-019-0352-3 (PMC6430765; doi:10.1038/s42003-019-0352-3)
Supplement: Supplementary file 4 — Description of Additional Supplementary Files [file 42003_2019_352_MOESM4_ESM.docx]

**Description of Additional Supplementary Files**

**File Name**: Supplementary Data 1

**Description:** The median value (in log2 scale) of gene expression (RSEM) in HPV-positive and virus-negative HNSC samples, EBV-positive and virus-negative STAD samples. We also show the difference between the two median values in third column and the calculated FDRs from Benjamini and Hochberg correction.
